# DNA Barcoding Reveals High Cryptic Diversity in the North Eurasian *Moina* Species (Crustacea: Cladocera)

**DOI:** 10.1371/journal.pone.0161737

**Published:** 2016-08-24

**Authors:** Eugeniya I. Bekker, Dmitry P. Karabanov, Yan R. Galimov, Alexey A. Kotov

**Affiliations:** 1 Laboratory of Aquatic Ecology and Invasions, A. N. Severtsov Institute of Ecology and Evolution of Russian Academy of Sciences, Moscow, Russia; 2 Laboratory of Fish Ecology, I. D. Papanin Institute for Biology of Inland Waters of Russian Academy of Sciences, Borok, Yaroslavl Area, Russia; 3 Laboratory of Experimental Embryology, Koltzov Institute of Developmental Biology of Russian Academy of Sciences, Moscow, Russia; University of Innsbruck, AUSTRIA

## Abstract

Species of the genus *Moina* Baird (Cladocera: Moinidae) often dominate freshwater crustacean communities in temporary water bodies. Several species of *Moina* are used as food for fish larvae in aquaculture, as bioindicators in toxicological studies, and as common subjects for physiological studies. The aim of this paper is to estimate biodiversity of *Moina* in northern Eurasia using the standard DNA barcoding approach based on the cytochrome c oxidase subunit I (*COI*) gene. We analysed 160 newly obtained and 157 existing *COI* sequences, and found evidence for 21 phylogroups of *Moina*, some of which were detected here for the first time. Our study confirmed the opinion that the actual species diversity of cladocerans is several times higher than is presently accepted. Our results also indicated that *Moina* has the second richest species diversity among the cladoceran genera (with only *Daphnia* O. F. Mueller having a greater diversity of species). Our study strongly supports division of *Moina* into two faunistic groups: European-Western Siberian and Eastern Siberian-Far Eastern, with a transitional zone at the Yenisey River basin (Eastern Siberia). Here, we refrain from taxonomic descriptions of new species, as this requires a thorough morphological and taxonomic study for each putative taxon.

## Introduction

Cladocerans, or water fleas, are microcrustaceans that often play important roles in aquatic food webs [1, 2]. The typical life cycle of cladocerans involves cyclical parthenogenesis and the production of resting eggs. These propagules are protected from unfavorable environmental conditions and are dispersed by other animals such as water birds [1, 3–5]. The vagility of cladocerans has contributed to an historical misconception that most species are "cosmopolitan" in their geographic distributions [6–10]. However, David Frey [11–12] provided morphological and biogeographic evidence that many cladocerans, in spite of their dispersal ability, have regional distributions.

Subsequent morphological studies have also found evidence that widely distributed taxa in many different groups of Cladocera are composed of a series of locally distributed biological species [12–16]. Early molecular studies that dealt mostly with the genus *Daphnia* O. F. Mueller [17–23], and much less with other groups [24–30] also supported regionalism. These molecular studies confirmed only a few near cosmopolitan cladoceran species–in these cases distribution patterns were affected by anthropogenic introductions [23].

The number of publications demonstrating cryptic species in various invertebrate taxa increased after applying DNA barcoding (i.e. use of short gene sequences for species identification [31]). In invertebrates, a fragment of the mitochondrial *COI* gene was found to be an informative molecular marker for such studies [32–33]. *COI* data were successfully used for taxon delimitation in cladocerans as well [34–36]. In a few cases, detailed morphological analysis of reconstructed genetic clades allowed descriptions of some new taxa [37–40]. In most cases cryptic lineages still await formal taxonomic recognition.

Temporary and semi-temporary water bodies can have unique fauna [5, 41–42] but remain poorly studied. In such localities, species of the genus *Moina* Baird are often dominant among freshwater microscopic animals [43–44]. By virtue of very high reproduction rates, some moinids are used in commercial aquaculture as food for fish larvae [1, 43]. Moreover, some species of the genus *Moina* are used as the "standard" test objects in toxicological and in physiological studies [45–47]. Still, the genus has received little attention by taxonomists [48–66] since the revisions of Goulden [43] and Smirnov [44]. At the same time, it is known that the morphological variability of moinid populations is high and the genus taxonomy is not completely resolved–some taxa are in fact represented by groups of cryptic species [44, 67, 68]. The first molecular study of species boundaries in *Moina* was performed by Petrusek et al. [67] who demonstrated that populations of *M*. cf. *micrura* from Europe and Australia belong to two different biological species, while populations of *M*. *macrocopa* from Czech Republic and Uganda presumably belong to the same species. Tatsuta et al. [69] isolated a set of microsatellite markers for *Moina macrocopa* suitable "for assessing cryptic genetic structure", but these markers were never used in any subsequent studies. The DNA barcoding studies of the genus were started by Elías-Gutiérrez et al. [35], followed by Jeffrey et al. [36], Prosser et al. [70] and Dumont et al. [65]. Finally, a comprehensive study of the *Moina brachiata* group in Hungary was made by Nédli et al. [42]. Based on *COI* and *16S* diversity, the latter authors found four cryptic species in Hungary alone. Greater geographic sampling is warranted to better assess the cryptic diversity of the genus.

Moinids contain over 25 named species [65, 71], and it is presently difficult to make a study with all of these taxa. At the same time, it is known that moinids avoid Arctic/Subarctic area and are rare in adjoined boreal territories [43–44, 67]. The number of taxa in the northern portion of Eurasia is presumably not that high, and the study of this region can be used as a starting point for the global assessment of biodiversity. The aim of this paper is to investigate the biodiversity of *Moina* of northern Eurasia using *COI* gene.

## Materials and Methods

### Field collection

Field collection in Russia was carried out by our team or by our colleagues as part of a governmental project "Ecology and biodiversity of aquatic ecosystems and invasions of alien species" (№ 0109-2014-0008), with governmental permission to collect samples from public property. Sampling in the natural reserves of Russia was conducted with special permission of their directors (V.E. Kirilyuk, the Director of Daursky Biosphere Reserve, Zabaikalsky Territory and Yu.P. Suschitsky, the Director of State Khankaisky Biosphere Reserve, Primorsky Territory). All collected samples were listed in special reports to the administration of the reserves. Verbal permissions to collect in private farm ponds were obtained from local owners. Mongolian samples were taken by the Joint Russian-Mongolian Complex Biological Expedition with permission of the Ministry of Nature, Environment and Tourism of Mongolia. Some samples from Hungary, Ukraine, Kazakhstan and China were provided by our colleagues having permissions to collect them due to their activity as hydrobiologists in governmental institutes in their countries. The field studies did not involve endangered or protected species.

Specimens were collected by plankton nets with diameter of 20–40 cm and mesh size of 30–50 μm, and rectangular dip nets of same mesh size with widht of 0.2–0.3 m, handle length of 0.5–2 m, and preserved in 90–96% alcohol. Before the start of the genetic studies, each specimen was preliminarly identified based on its morphology [43–44, 72].

### DNA sequencing

Genomic DNA was extracted using Wizard Genomic DNA Purification Kit (Promega Corporation, Madison, WI, USA) according to the manufacturer’s instructions. The polymerase chain reaction (PCR) was used to amplify a 710 bp fragment of the 5' region of the COI gene using the redesigned universal primer pair jgHCO2198 and jgLCO1490 [73], tailed with the M13 sequence [74], which was necessary due to a high level of degeneracy of the primers [75, 76]. These primers were used for amplification of all ingroup and outgroups. The 25-μL PCR reaction consisted of 2 μL of genomic DNA, 8.5 μL of double-distilled H_2_O, 1 μL of each primer (10 mM) and 5 μL PCR 5x Taq ScreenMix-HS (Evrogen, Moscow, Russia). PCR conditions used for the COI amplification were as following: 1 cycle of 5 min at 95°C, 40 cycles of 60 s at 95°C (denaturation), 90 s at 42°C (annealing) and 90 s at 72°C (extension), followed by 1 cycle of 6 min at 72°C. The PCR products were electrophoresed in a 1.5% agarose gel stained with ethidium bromide and were visualized under UV light. A 0.1–3 kb DNA ladder (SibEnzyme, Novosibirsk, Russia) was used for the estimation of the amplicon length. The obtained PCR products were reprecipitated at room temperature by adding ethanol to the final concentration of 70% and ammonium acetate to the final concentration of 125 mM. The DNA precipitate was washed with 70% ethanol, dried, and dissolved in distilled water. About 0.3 pmol of the PCR product and 3.2 pmol of the relevant primer were used for the sequencing reaction. Each PCR product was sequenced bi-directionally using an ABI 3730 DNA Analyzer with ABI PRISM BigDye Terminator v. 3.1 sequencing kit (Applied Biosystems, USA). A single consensus sequence was assembled using the forward and reverse sequences using CodonCode Aligner v. 6.0.2 (CodonCode Corp, USA). DNA sequences were submitted to the NCBI GenBank database (accession numbers KX168502-KX168592) (S1 Table).

### Analysis of genetic divergence

The authenticity of the sequences was verified by BLAST comparisons. Bidirectional sequencing of all nucleotide sequences was proofread, edited and assembled in UGENE v. 1.22.0 package [77]. We used *COI* sequences from previous studies [33–36, 42] and some directly deposited sequences available from GenBank (S2 Table) and aligned them with our original sequences. Several taxa of the Daphniidae, the family which is a sister group to Moinidae [4], were used as outgroups. The sequence of "*Moina* sp." (GenBank KC617696.1 [69]) was excluded as not belonging to *Moina*.

The DNA sequences were first automatically aligned using the ClustalX algorithm [78] using default options and then manually edited. The primer sequences were removed from the alignment prior to any further analysis, which therefore was based on the 657 bp fragment. To analyse the polymorphism among *Moina* populations, the following parameters were evaluated: the number of polymorphic sites (S), number of haplotypes (h), haplotype diversity (Hd), nucleotide diversity (Pi), average number of nucleotide differences (k) and Tajima's neutrality test (D). All calculations were performed using DnaSP v. 5.1 [79] and MEGA v.6 [80].

The best-fitting models of nucleotide substitution were selected in jModelTest 2.1.7 [81] based on the likelihood scores for 88 different models and the Akaike information criterion [82]. The best model was general time reversible [83] with a gamma distribution and proportion of invariable sites (GTR+G+I).

*Ceriodapnia* and *Daphnia* species were employed as suitable outgroups in phylogenetic tree reconstruction. The maximum likelihood (ML) analysis (which used the GTR+G+I evolutionary model), minimum evolution (ME) method and maximum parsimony phylogeny reconstruction was performed with MEGA v.6 and bootstrap resampled 1000 times. Bayesian analyses (BI) were performed in MrBayes v.3.2.6 [84]. Four independent Markov chain Monte Carlo (MCMC) analyses were run simultaneously for 5 million generations and sampled every 1000 generations. The first 25% of the generations were discarded as the burn-in and a 50% majority rule consensus tree was calculated from the remaining trees. The mean genetic sequence divergence between major phylogroups was calculated in MEGA v.6 using Tamura 3-parameter model [85] and gamma rates distribution with the shape parameter estimated by jModelTest and with pairwise deletion of gaps and 10000 bootstrap resampled. Substitution pattern and rates were also estimated under the Tamura model [82].

Species clusters were collapsed using the FigTree Version 1.4.2 Collapse module.

## Results

The overall sequencing success rate was about 70%. The high failure rate was mainly due to problematic clades, i.e. *Moina* cf. *micrura* from the Far East of Russia (presumably *M*. *chankensis*), for which not a single sequencing was successful. PCR failure may have resulted from sequence mismatches of the template at the primer binding site. The alignment contained 160 original and 157 NCBI GenBank *COI* sequences. We identified (for *Moina* only) 635 conserved sites (excluding sites with gaps/missing data), including 386 invariable sites, 249 variable sites, 397 number of mutations, 6 singleton variable sites and 243 parsimony-informative sites. The A+T content (67%) was higher than the G+C content (36%), similarly to other cladocerans and all invertebrates (see records in Wang et al. [86]). The estimated transition/transversion bias t is 3.27. The levels of genetic differentiation (mean between-clade Tamura 3-parameter distance) of revealed lineages ranged from 3.9% to 28.1% (Table 1 and S3 Table).

**Table 1:** Polymorphism of the COI gene mtDNA in *Moina* populations.

Phylogroup number	Phylogroup name	n	S	h	Hd	Pi	k	Tajima’s D		Haplotype numbers
								D	P	
1	*Moina* cf. *brachiata* clade A	29	21	13	0.936	0.008	5.08	-0.329	>0.10	H_24, H_25, H_36, H_48, H_49, H_50, H_51, H_52, H_54, H_58, H_77, H_78, H_79
2	*Moina* cf. *brachiata* clade B	24	5	5	0.728	0.002	1.09	-0.521	>0.10	H_01, H_06, H_15, H_16, H_38
3	*Moina* cf. brachiata clade C	10	2	3	0.511	0.001	0.55	-0.690	>0.10	H_03, H_04, H_46
4	*Moina* cf. *brachiata* clade D	14	13	6	0.857	0.007	4.68	0.583	>0.10	H_17, H_23, H_39, H_41, H_44, H_69
5	*Moina* cf. *brachiata* clade E	38	14	11	0.785	0.005	3.11	-0.205	>0.10	H_68, H_70, H_71, H_72, H_73, H_74, H_75, H_76, H_80, H_81, H_84
6	*Moina* cf. *brachiata* clade F	5	3	3	0.700	0.003	1.60	0.699	>0.10	H_09, H_82, H_83
7	*Moina* cf. *brachiata* clade G	8	18	4	0.643	0.007	4.67	-1.687	<0.05	H_26, H_27, H_28, H_47
8	*Moina* cf. *micrura* clade H	6	3	3	0.600	0.002	1.40	0.338	>0.10	H_02, H_08, H_53
9	*Moina* cf. *micrura* clade I	13	6	4	0.756	0.004	2.43	0.959	>0.10	H_22, H_43, H_92, H_93
10	*Moina* cf. *micrura* clade J	2	0	1	-	-	-	-	-	H_56
11	*Moina* cf. *micrura* 5 Mexico	17	0	1	-	-	-	-	-	H_89
12	*Moina* cf. *micrura* 4 Mexico	4	0	1	-	-	-	-	-	H_94
13	*Moina* cf. *micrura* 1 Mexico	19	8	6	0.713	0.004	2.46	0.267	>0.10	H_60, H_61, H_62, H_63, H_64, H_65
14	*Moina* sp. Canada	1	-	1	-	-	-	-	-	H_59
15	*Moina* cf. *micrura* 2 Mexico	35	5	3	0.113	0.001	0.39	-1.775	>0.05	H_66, H_87, H_88
16	*Moina lipini*	7	5	3	0.714	0.003	1.714	-0.792	>0.10	H_20, H_21, H_45
17	*Moina macrocopa amiricana*	9	7	3	0.639	0.005	3.11	0.927	>0.10	H_67, H_90, H_91
18	*Moina* cf. *macrocopa* clade L	6	3	2	0.600	0.003	1.80	1.909	>0.05	H_31, H_32
19	*Moina macrocopa macrocopa*	36	33	11	0.852	0.017	11.61	1.471	>0.10	H_11, H_13, H_14, H_18, H_19, H_37, H_40, H_42, H_57, H_85, H_86
20	*Moina* cf. *salina* clade N	9	13	6	0.833	0.004	2.88	-1.889	<0.05	H_29, H_30, H_33, H_34, H_35, H_95
21	*Moina* cf. *salina* clade O	13	12	5	0.808	0.006	3.97	0.113	>0.10	H_05, H_07, H_10, H_12, H_55
Total		305	249	94						95

n—sample size, S—number of polymorphic sites, h—number of haplotypes, Hd—haplotype diversity, Pi—nucleotide diversity, k—average number of nucleotide differences, D—Tajima's neutrality test, P—statistical significance Tajima’s D.

No indels or stop codons were observed in the alignment visually or by using the ORF module of the uGene program. The mean intraspecific divergence was 0.18, while the maximum intraspecific divergence was 0.28. In contrast, the average intraspecific divergence ranged from 0.04 to 0.13 (S3 Table).

The separation of taxa based on the analysis of a single mitochondrial gene (i.e. *COI*) could be be complicated by the existence of functionless nuclear copies or pseudogenes [87, 88, 89, 90]. In the case of *Moina* we probably studied coding mitochondrial *COI* sequences. We found no evidence for pseudogenes such as stop codons and indels (see above). Also, a previous study of this genus revealed congruence of the *COI* phylogeny with that based on *16S* and nuclear markers [42].

**Fig 1:**
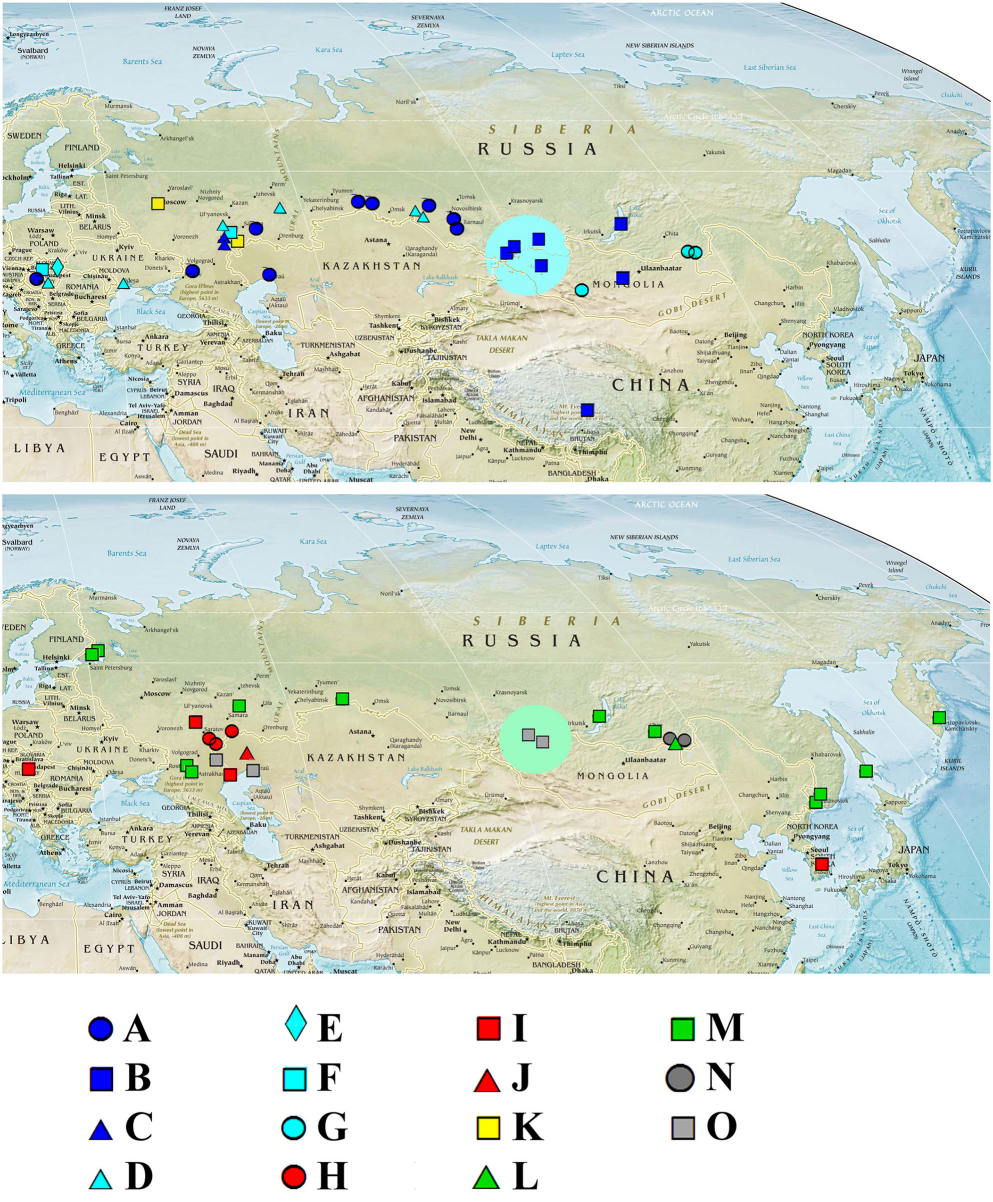
Eurasian sites from which diversity of the genus *Moina* has been analysed. Upper panel—*brachiata* (blue symbols) and *lipini* (yellow symbols) groups, bottom panel—*macrocopa* (green symbols), *micrura* (red symbols) and *salina* (grey symbols) groups. A-M—15 clades revealed within Eurasian range. The initial map is from CIA public domain: https://www.cia.gov/library/publications/resources/the-world-factbook/docs/refmaps.html

**Fig 2:**
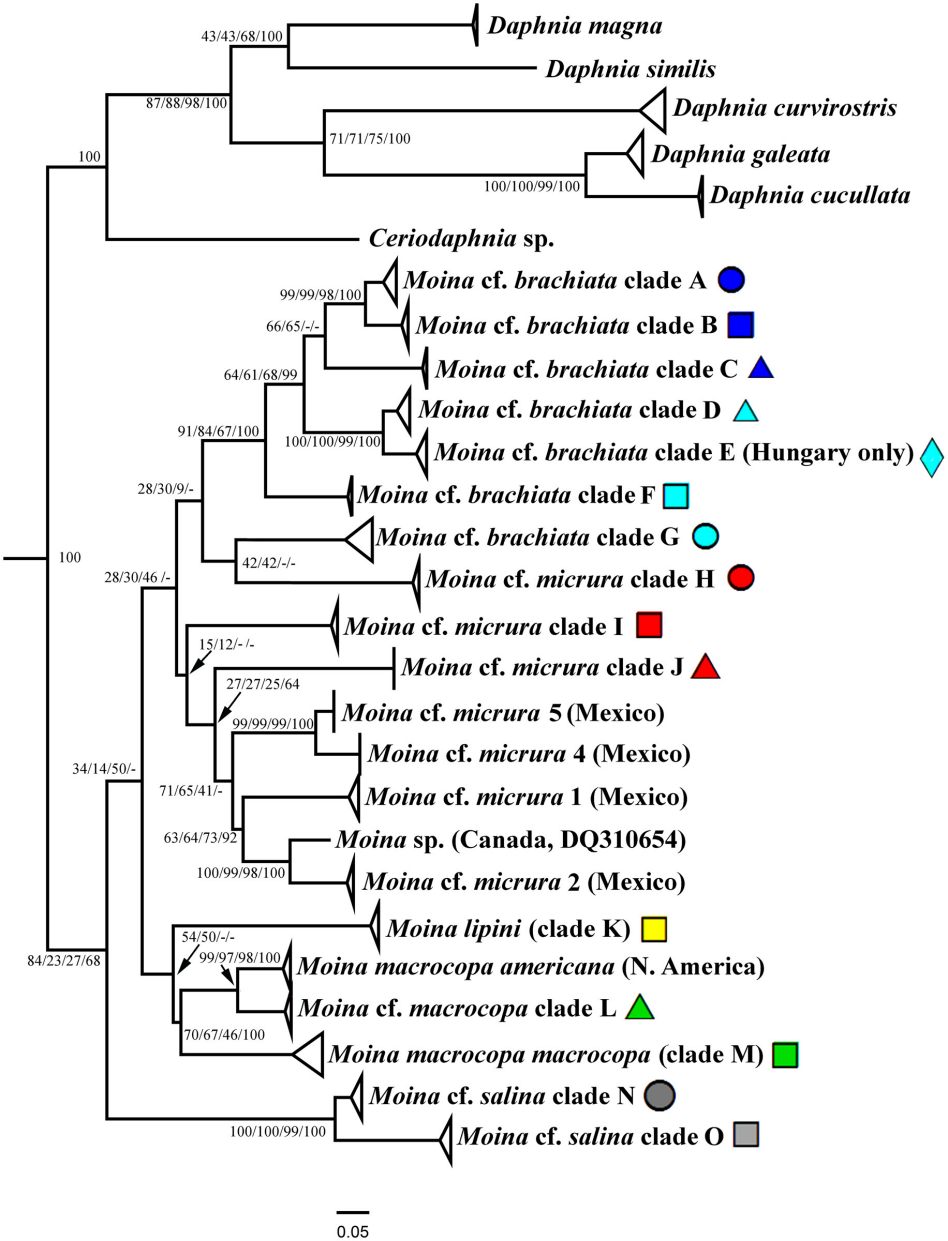
Maximum likehood tree representing the diversity among phylogroups of *Moina*. Symbols near Eurasian taxa correcpond to those in Fig 1. The support values of individual nodes are based on different variants of phylogenetic analysis: ML / ME / MP / BI. See Figs 3 and 4 for support of terminal clades.

**Fig 3:**
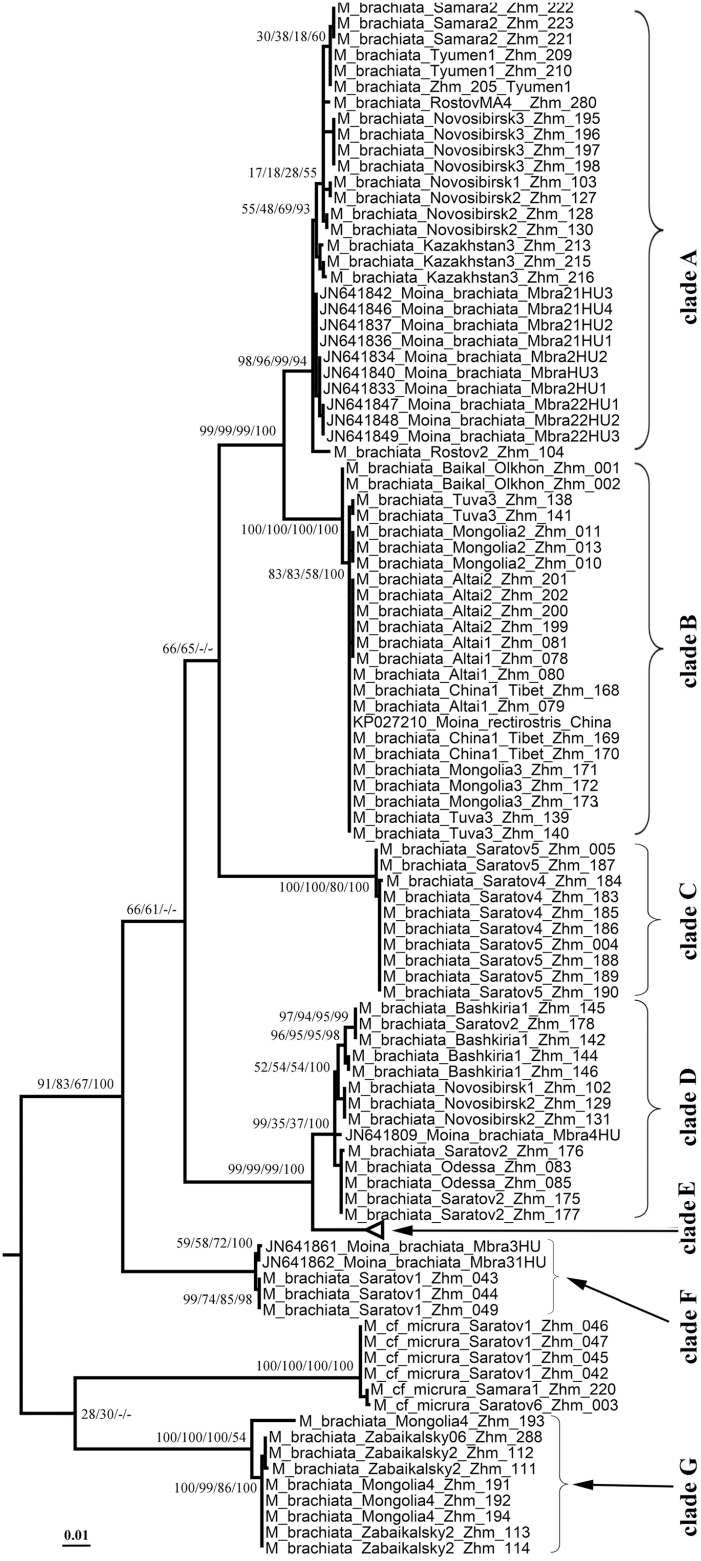
Uncollapsed portion of the tree represented in Fig 2 for the *brachiata*-like taxa with support of terminal clades added.

**Fig 4:**
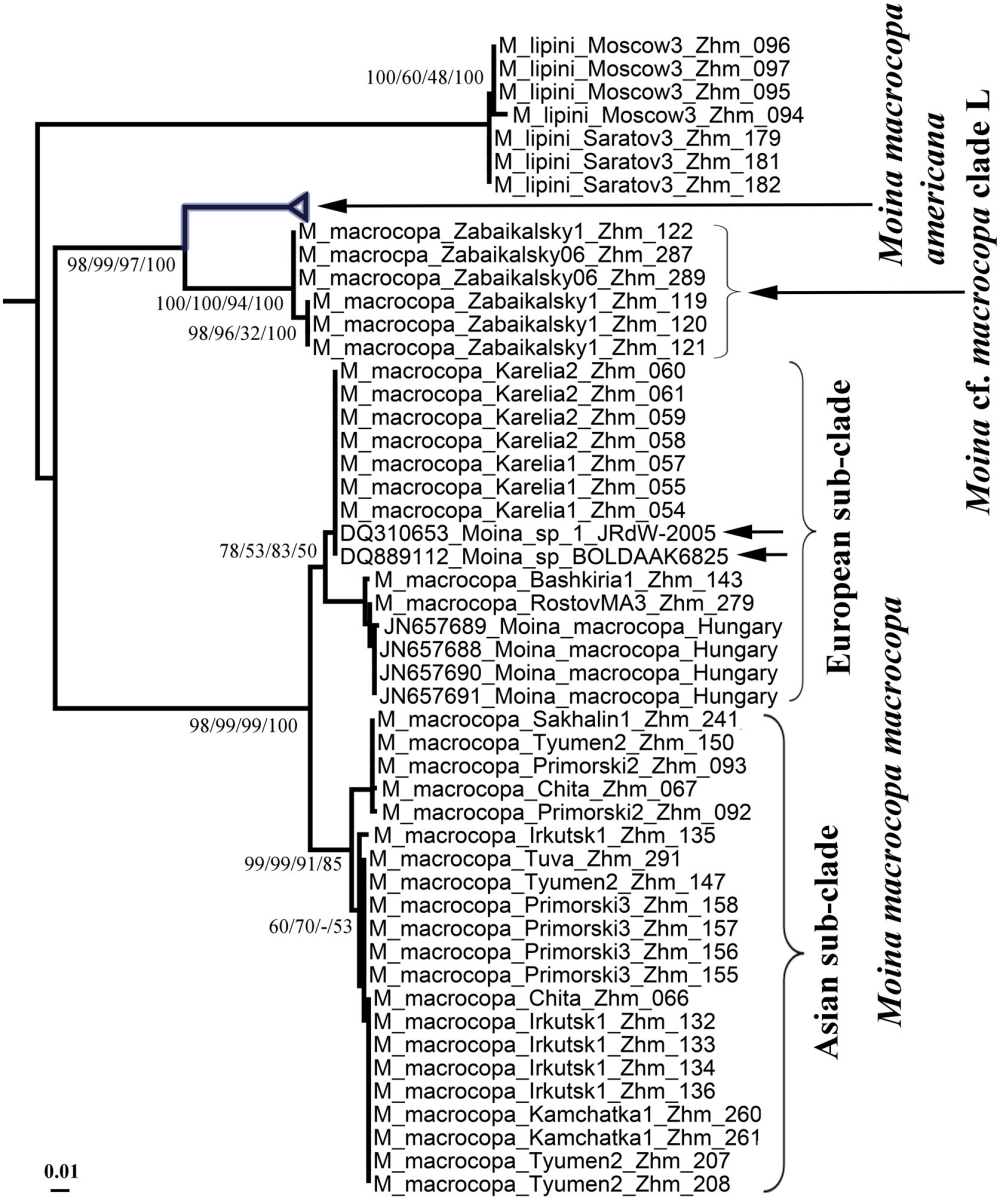
Uncollapsed portion of the tree represented in Fig 2 for the *macrocopa*-like taxa. Arrows indicate two sequences of *Moina macrocopa macrocopa* of presumably Canadian origin.

Original sequences together with the GenBank sequences could be associated with 21 phyloclades in ML search. We numbered all clades from Eurasia from ML search by capital letters from A to O, while the North American clades have no special abbreviations here and minimally discussed in this paper. For the *brachiata*-like taxa we used our own numeration of clades, different from that by Nédli et al. [42]. In total, we differentiate six clades of the *brachiata*-like taxa (A-G, all from Eurasia), eight clades of the *micrura*-like taxa (five North American clades and three Eurasian clades, H-J), a single clade for *Moina lipini* (K), three clades of the *macrocopa*-like taxa (single North American and two Eurasian clades, L-M), and two clades of the *salina*-like taxa (both from Eurasia, N-O) (Figs 1–4).

Trees constructed by different methods are in general congruent, but support of different clades is somewhat different in different types of the analysis. The following clades have a strong support in any types of the analysis: A + B; D + E; *M*. cf. *micrura* 5 + *M*. cf. *micrura* 4; *M*. sp. + *M*. cf. *micrura* 2. Few clades moderately supported in ML were not supported in BI (A+B+C; *M*. *c*f. *micrura* 5 + *M*. cf. *micrura* 4 + *M*. cf. *micrura* 1 + *M*. sp. + *M*. cf. *micrura* 2; K + L + M). In contrast, some other clades with a strong support in BI have worse support in ML (A + B + C + D + E; *M*. cf. *micrura* 1 + *M*. sp. + *M*. cf. *micrura* 2; *M*. *macrocopa americana* + L + M + N). The support of branches in ME and MP was in general similar with that in ML.

As it is marked below, statistical support for the deep branches is low in all searches, the grouping of the deeper clades preliminarly support separation of three super-clades: (1) the *brachiata*-*micrura* group, (2) the *macrocopa-lipini* group, (3) the *salina* group. Note that the *brachiata* and *micrura*-like clades do not form monophyletic groups in our tree, see Discussion.

### The *brachiata*-like clades (Fig 3)

Clade A is present in Hungary [42], southern portion of European Russia and Western Siberia up to Novosibirs Area (up to about 79°E). Clade B is found in west-southern portion of Eastern Siberia, Mongolia and Chinese Tibet. One more sequence from China present in the GenBank was attributed to this clade, but there is no detailed information on the locality from where is the speciemen originated. It is obvious that this clade is widely distributed in China, from South to the North of the country. Clade C is found only in three close localities in Saratov Area (south portion of European Russia). Clade D is present in Hungary [42], Ukraine and southern portion of European Russia. Clade E is found in Hungary only, where it is the most common phylogroup [42], but it is fully absent in European Russia, well-represented by our samples. Clade F is found in Hungary [42] and south portion of European Russia. Clade G is present in Mongolia and Transbaikalian portion of Eastern Siberia.

### The *micrura*-like clades

Clade H is found only in southern portion of European Russia. Clade I is present in Hungary [42], southern portion of European Russia, Kazakhstan. A single population is present in South Korea (sequence of Prosser et al., 2013), but it could be a case of a human-mediated introduction, see below. Clade J is represented by two specimens from s single population in Kazakhstan. Five other clades are found in North America [35–36].

### The *lipini* clade

Clade K is found in central and southern portion of European Russia.

### The *macrocopa*-like clades (Fig 4)

Clade L is found only in two localities in Transbaikalian Area, Eastern Siberia, we can assume that this is a locally distributed phylogroup. Clade M (*Moina macrocopa macrocopa*) is widely distributed, it was detected in Hungary [42], north and south portions of European Russia, Western Siberia, Eastern Siberia, Far East of Russia and even Kamchatka Peninsula. Two sequences from this clade [33, 34] are known from the GenBank and presumably belong to Canadian populations (but it needs to be specially checked, see below). Remarkably, there are two geographical sub-clades within the clade M in the ML tree, European-Western Siberian and Easter Siberian-Far Eastern, but support of these clades is low.

## Discussion

### Cryptic diversity in *Moina* from Palaearctic

Hebert et al. [31–32] proposed to consider two clades as distinct species if the divergence between them in *COI* sequences is greater than 3%, lower values (0.7–2.2%) suggest a recent divergence of a clade. Here we accept this approach for the species delimitation, keeping in mind possible differences in the mutation rates among different groups.

Our study confirms the opinion that the real diversity of the cladocerans is several times higher than is accepted now [2, 91]. We found several cryptic species complexes within *M*. *macrocopa*, *M*. *brachiata*, *M*. *micrura* and *M*. *salina*, species confirming preliminary conclusions of Petrusek et al. [67] and Nédli et al. [42]. In comparison to previous publications, we significantly increased the number of revealed phylogroups which presumably correspond to biological species. It needs to be pointed out that our study has covered only the northern half of Eurasia, while the whole southern half of the continent is unexplored, as well as other continents (except of some portions of North America). In addition, only a small portion of the *Moina* species were investigated, while *M*. *weismanni* (common in different regions of Eurasia [43, 60, 54, 56, 57], *M*. *reticulata* (common in the Neotropics [43]), several Australian endemics [92], several newly described endemics with a very particular morphology [62, 64, 66], *M*. cf. *micrura* in Far East (for which we had samples, but, for unknown reasons, all PRCs were not successive), and many other species were out of our attention due to absence of a DNA-available material. Petrusek et al. [93] proposed that "species-rich groups that have mostly escaped the interest of molecular taxonomists and molecular ecologists (e.g., cladoceran genera *Ceriodaphnia*, *Diaphanosoma*, *Moina*, most members of the cladoceran families Chydoridae and Macrothricidae, as well as a wide range of freshwater copepods) hide enormous diversity that remains to be discovered". Now we can confidently assume that the number of moinid species is comparable to that in *Daphnia*, meaning that *Moina* is one of the largest genera of the Cladocera. Even after the splitting of *Alona* Baird into many species [16, 94], *Moina* could be regarded as the second most species rich Cladoceran genus, after *Daphnia* O. F. Mueller.

Our study strongly supports the non-cosmopolitism concept [11–12]. Among studied taxa, only a single taxon is presumably present both in Europe and North America, *M*. *macrocopa macrocopa* (clade M). However, this conclusion is based on two sequences of dubious, presumably Canadian, origin (marked by arrows in Fig 4). They originated from the publication [33–34] with no exact information on the localities. Origin of this population could be explained by a human-mediated introduction. Even both sequenced specimens could be originated from a laboratory culture of European origin instead of a Canadian locality. At the same time, these specimens could be really from a Canadian population. Colonization of New World by *M*. *macrocopa macrocopa* as a result of human-mediated introduction was documented several times by morphological taxonomists [63, 95, 96, 97].

There are no other species shared between Eurasia and North America, or/and any other continents. It is known that at least the *micrura*- and the *macrocopa* species groups have taxa on different continents, including even Australia. Such multi-continental pattern could be regarded as a confirmation of ancient, possibly Mesozoic, differentiation within these species groups that occurred before the continental break up, similarly to the subgenera and some species groups of *Daphnia* [98] or *Simocephalus* [30]. It is known that *Moina* diversity exploded in the Mesozoic, since ephippia, possibly belonging to this genus, were found in the Mongolian locality from the Jurassic/Cretaceous boundary [4, 99]. In contrast, a differentiation of the *brachiata*-like taxa presumably took place in Eurasia already after break up of the continents, as no *brachiata*-like populations were found in New World and Australia [43].

For the moment, the exact geographic boundaries of phylogroup distributions remain unknown, but many of the phylogroups provisionally seem to be endemics of some relatively local territories. Such situation does not seem to be exceptional among different groups of the cladocerans [86].

There is a general rule in the distribution of different phylogroups within different species groups: except of widely distributed *M*. *macrocopa macrocopa*, they could be subdivided into (1) European-Western Siberian (A, C, D, E, F, H, I, J, K, O) and (2) Eastern Siberian-Far Eastern (B, G, L, N). The exact boundary between the former and the latter is somewhat different in *brachiata*, *macrocopa*- and *salina*-groups. In general, we can draw a transitional zone between the European-Western Siberian and Eastern Siberian-Far Eastern phylogroups (= faunistic complexes) in the Yenisey River basin (highlighted in Fig 1A in blue and in Fig 1B in green). Preliminary analysis of the *COI* divergence in different group of *Moina* shows that this gene has a strong phylogeographic signal, but more sequences are necessary for good haplotype networks and phylogeographic conclusions based on them.

The study of the *micrura*-like taxa must be continued, because for some reasons we do not have genetic information on the populations from Eastern Siberia and Far East of Russia. There is a chance that a single population of *M*. *micrura* from clade I (sequences of [69]) appeared in South Korean reservoir due to a human-mediated introduction.

The very high lineage richness of the *Moina* in Carpatian and Transbaikalian regions suggests that these boreal regions could be important centers of diversity for the genus. Presumably, they could be associated with some refugia, where some lineages survived well-known cold and arid conditions of the Pleistocene, but further studies are necessary to understand exact pattern of the species distribution in Eurasia.

### Consequences for the moinid studies

We found that several "traditional" taxa in Eurasia are represented by series of separate mitochondrial phylogroups. But there are some well-known limitations of the barcoding approach. For example, a hybridisation and nuclear introgression were previously demonstrated for some lineages of *Daphnia* [100–105]. Such phenomena could make the differentiation of biological species in the moinids much more complicated than barcoding diagnosing. But we agree with the opinion, that "ultimately, the responsibility of accurate identification of animal specimens rests with the researchers who determine species’ identity using a host of morphological characters" [105].

In the case of *Moina*, even the preliminary naming of such taxa is presently impossible. A checklist of all "species-group nominal taxa" sensu ICZN [106] composed by AAK (it is partly represented in [70]) contains 78 formal names which could be attributed to the genus *Moina*. Among them, there are four taxa from the *brachiata*-group, 15 taxa from the *macrocopa*-group, 11 taxa from the *micrura*-group, and four taxa from the *salina*-group (Table 2).

**Table 2:** List of formal taxa presumably belonging to four large groups of *Moina*. Taxa are given in original spelling and ordered in each column chronologically.

The *brachiata*-group	The *macrocopa*-group	The *micrura*-group	The *salina*-group
*Monoculus brachiatus* Jurine, 1820	*Daphnia macrocopus* Straus, 1820	*Moina micrura* Kurz, 1875	*Moina salina* Daday, 1888
*Moina lilljeborgii* Schödler, 1877	*Moina flagellata* Hudendorff, 1876	*Moina weberi* Richard, 1891	*Moina mongolica* Daday, 1901
*Macrothrix magnantennula* Cosmovici, 1900	*Moina fischeri* Hellich, 1877	*Moina makrophthalma* Stingelin, 1913	*Moina microphthalma* Sаrs, 1903
*Moina rectirostris* var. *caucasica* Schikleev, 1930	*Moina paradoxa* Weismann, 1880	*Moina macrocopa* var. *brevicaudata* Вär, 1924	*Moina salinarum* Gurneу, 1909
	*Moina bánffyi* Daday, 1883	*Moina dubia lacustris* Rammner, 1931	
	*Moina azorica* Moniez, 1888	*Moina dubia macrocephala* Rammner, 1933	
	*Moina paradoxa* var. *japonica* Ishikawa, 1896	*Moina latidens* Brehm, 1933	
	*Moina rectirostris* var. *Casañi* Arévalo, 1920	*Moina dubia* var. *parva* Rammner in Jenkin, 1934	
	*Moina esau* Brehm, 1936	*Moina dubia* var. *baringoensis* Jenkin, 1934	
	*Moina esau* var. *dschirofti* Hemsen, 1952	*Moina chankensis* Uénо, 1939	
	*Moina ganapatii* Brehm, 1963	*Moina dodhui* Rane, 1987	
	*Moina macrocopa americana* Goulden, 1968		
	*Moina kazsabi* Forró, 1988		
	*Moina gouldeni* Mirabdullaev, 1993		

The authors of these taxa in their original descriptions proposed some diagnostic characters, but most of these "diagnostic traits" seem to be dubious, at least, their value needs to be re-evaluated. Due to this Goulden [43] and then Smirnov [44] regarded all such taxa as junior synonyms of previously described species. The situation was not improved after these two global revisions, and more new taxa with such subjective diagnostic characters were added (see Table 2). In general, the problems in the *Moina* taxonomy are quite similar to those in *Daphnia*, see the critical discussion by Kotov [107]. As in the case of *Daphnia*, we believe that an accurate taxonomic revision based on male and ephippial female characters [103] could improve the taxonomy of *Moina*.

The authors who studied morphology of the moinids were victims of the so-called "sharp" diagnostic characters and the so-called comb-shaped keys [108]. These keys [43–44, 71] are used now for determination of the moinids. They immediately identified a specimen with a strong pecten of teeth on the postabdominal claw as *M*. *brachiata*, a specimen with a specific (having tooth-like setules) seta on the penultimate segment of limb I–as *M*. *macrocopa*, and a specimen having no seta on the penultimate segment–as *M*. *salina*. No further investigations of other morphological traits were conducted. As a result, to date we do not have an adequate information on the morphological differences between *brachiata*, *macrocopa* and *salina*-like populations from different regions. For example, local *M*. *salina*-like clade from Mongolia and its vicinities could belong to *M*. *mongolica* Daday, but may be a new species.

Interestingly, new lineages of *Moina* were found not only in poorly studied regions like Eastern Siberia (with only few studies concerning the genus reported from the area), but also in the southern portion of European Russia, in which numerous works on the genus have been conducted [109, 110]. Previous ecological works on *Moina* from Eastern Europe and other territories [52, 111, 112] in reality dealt with a number of taxa. As a result, the information on biology of separate "species" in previous summarizing publications [113, 114] is a chimerical mix composed from data on different taxa with possible differences in their ecological preferences.

### Short comments on the moinid system

Before 2010, it was accepted that the family Moinidae includes two genera: *Moina* Baird and *Moinodaphnia* Herrick [43, 44, 71]. Hudec [56] proposed to subdivide the genus into two subgenera, *Moina* s.str. and *Moina* (*Exomoina*) Hudec, the latter taxon unites the species with two eggs in the ephippium and a large "exopodite" on male limb I. Hudec [56] studied specimens from Middle Europe only: *M*. *brachiata*, *M*. *micrura*, *M*. *weismanni* were included to the former subgenus; *M*. *macrocopa* and *M*. *ephemeralis* (the latter is a presumable junior synonym of *M*. *lipini*)—to the latter subgenus; *M*. *salina* was not placed to any subgenera. This subdivision has a great defect: very numerous Non-European taxa were not classified by the author, and still we have no ideas on their subgeneric status according to the scheme of Hudec [56]. Afterwards, Dumont et al. [65] even proposed to rise the status of *Exomoina* up to a separate genus and described one genus more, *Micromoina* Dumont, Rietzler et Kalapothakis, i.e. referring to a small *COI* tree. In our view, this step is wrong. Morphological differences between moinid species are less expressed than those between different species of *Daphnia*, but nobody tried to subdivide the latter genus into a series of genera. The divergence in the *COI* sequences in *Moina* is comparable with that between the genera of Daphniidae (Fig 2), but a *COI* tree could not be regarded as strong evidence for a taxonomic scheme. Separation of *Moina* into several genera will lead to taxonomic confusion, i.e. a difficulty in assigning names to specimens of non-European moinids (with undescribed ephippium and male) even up to genus level.

Even the subgenera of *Moina* by Hudec [56] are problematic according to our data. The grouping of the subgenus *Moina* (*Exomoina*) (clades K, L, M plus *M*. *macrocopa americana*), with two obvious morphological synapomorphies listed by Hudec [56], has moderate support in some variants of our phylogenetic analysis. But *Moina* s.str. clade (clades A-J plus clades of North American *M*. *micrura*) has insufficient support for any discussions of its monophyly. It is possible that this is a paraphyletic assemblage of some non-related taxa. At the same time, the separate status of *M*. *salina* group is obvious from our data. Therefore only new genetic and morphologic studies could result in an adequate moinid system.

## Conclusions

Our study unambiguously confirms the existence of many phylogroups of the genus *Moina* in Eurasia. Congruence of these groups with biological species needs to be confirmed by a phylogenetic study based on nuclear genes and morphological analysis with the aim to find adequate diagnostic characters. Such studies need to be accompanied by taxonomic efforts for a proper naming of all taxa, i.e. descriptions of those new for science.

## Supporting Information

S1 Table Complete list of sequences obtained in this study with information on locality, haplotype number and the GenBank accession number provided for each specimen. Clade designations correspond to those in other tables. AR in list of states = Autonomous Republic.
(DOC)

S2 Table Complete list of sequences from the GenBank that were used in our study. (DOC)

S3 Table Intra-group and inter-group genetic distances of the 21 groups involved 657 nucleotide sequences of *Moina*. Number of phylogroups correspond to thise in other tables. Standard error estimates are shown above the diagonal and were obtained by a bootstrap procedure (10000 replicates). Analyses were conducted using the Tamura 3-parameter model. The rate variation among sites was modeled with a gamma distribution. All ambiguous positions were removed for each sequence pair.
(DOC)

## References

[1] Dumont HJ, Negrea SV, Introduction to the class Branchiopoda. Leiden: Backhuys Publishers; 2002.

[2] Forró L, Korovchinsky NM, Kotov AA, Petrusek A. Global diversity of cladocerans (Cladocera; Crustacea) in freshwater. Hydrobiologia. 2008;595: 177–184.

[3] Proctor VW. Viability of Crustacean eggs recovered from ducks. Ecology. 1964;45: 656–658.

[4] Kotov AA. Morphology and phylogeny of the Anomopoda (Crustacea: Cladocera). Moscow: KMK Press 2013.

[5] Incagnone G, Marrone F, Barone R, Robba L, Naselli-Flores L. How do freshwater organisms cross the “dry ocean”? A review on passive dispersal and colonization processes with a special focus on temporary ponds. Hydrobiologia. 2014;750: 103–123.

[6] Darwin C. On the Origin of Species by Means of Natural Selection, or the Preservation of Favoured Races in the Struggle for Life. London: John Murray; 1859. PMC5184128 30164232

[7] Darwin C. On the dispersal of freshwater bivalves. Nature. 1882;15: 529–530.

[8] Brehm V. Apostillas zoogeograficas a varios trabajos del Prof. H. Gauthier, con un apéndice sobre las caracteristicas biogeograficas de algunos grupos de organismos dulciacuicolas. Publicationes del Instituto de Biologia Aplicada.1950;7: 83–130

[9] Brehm V. Sobre la microfauna de las aquas dulces de diversos paises extraeuropeos. Publicationes del Instituto de Biologia Aplicada. 1951;5: 83–100.

[10] Brehm V. Süsswasserfauna und Tiergeographie. Österreichische Zoologische Zeitschrift. 1955;6: 250–269.

[11] Frey DG. Questions concerning cosmopolitanism in Cladocera. Arch Hydrobiol. 1982;93: 484–502.

[12] Frey DG. The taxonomy and biogeography of the Cladocera. Hydrobiologia. 1987;145: 5–17.

[13] Frey DG. *Alona weinecki* Studer on the subantarctic islands, not *Alona rectangula* Sars (Chydoridae, Cladocera). Limnol Oceanogr. 1988;33: 1386–1411.

[14] Smirnov NN. A revision of the genus *Camptocercus* (Anomopoda, Chydoridae, Aloninae). Hydrobiologia. 1998;386: 63–83.

[15] Dumont HJ, Silva-Briano M. *Karualona* n.gen. (Anomopoda: Chydoridae), with a description of two new species, and a key to all known species. Hydrobiologia. 2000;435: 61–82.

[16] Van Damme K, Sinev AY, Dumont HJ. Separation of *Anthalona* gen.n. from *Alona* Baird, 1843 (Branchiopoda: Cladocera: Anomopoda): morphology and evolution of scraping stenothermic alonines. Zootaxa. 2011;2875: 1–64.

[17] Taylor DJ, Hebert PDN. Genetic assessment of species boundaries in the North American *Daphnia longispina* complex (Crustacea: Daphniidae). Zool J Linn Soc. 1994;110: 27–40.

[18] Taylor DJ, Finston TL, Hebert PDN. Biogeography of a widespread freshwater crustacean: Pseudocongruence and cryptic endemism in the North American *Daphnia laevis* complex. Evolution. 1998;52: 1648–1670. 28565305 10.1111/j.1558-5646.1998.tb02245.x

[19] Adamowicz SJ, Hebert PDN., Marinone MC. Species diversity and endemism in the *Daphnia* of Argentina: a genetic investigation. Zool J Linn Soc. 2004;140: 171–205.

[20] Adamowicz SJ, Petrusek A, Colbourne JK, Hebert PDN, Witt JDS. The scale of divergence: a phylogenetic appraisal of intercontinental allopatric speciation in a passively dispersed freshwater zooplankton genus. Mol Phylogenet Evol. 2009;50: 423–436. 10.1016/j.ympev.2008.11.026 19124080

[21] Petrusek A, Hobæk A, Nilssen JP, Skage M, Černý M, Brede N, Schwenk K. A taxonomic reappraisal of the European *Daphnia longispina* complex (Crustacea, Cladocera, Anomopoda). Zool Scr. 2008;37: 507–519.

[22] Mergeay J, Aguilera X, Declerck S, Petrusek A, Huyse T, De Meester L. The genetic legacy of polyploid Bolivian *Daphnia*: the tropical Andes as a source for the North and South American *D*. *pulicaria* complex. Mol Ecol. 2008;17: 1789–1800. 10.1111/j.1365-294X.2007.03679.x 18284570

[23] Crease TJ, Omilian AR, Costanzo KS, Taylor DJ. Transcontinental phylogeography of the *Daphnia pulex* species complex. PLoS ONE. 2012;7(10): e46620 10.1371/journal.pone.0046620 23056371 PMC3463573

[24] Haney RA, Taylor DJ. Testing paleolimnological predictions with molecular data: the origins of Holarctic *Eubosmina*. J Evol Biol. 2003;16: 871–882. 14635902 10.1046/j.1420-9101.2003.00594.x

[25] Belyaeva M, Taylor DJ. Cryptic species within the *Chydorus sphaericus* species complex (Crustacea: Cladocera) revealed by molecular markers and sexual stage morphology. Mol Phylogenet Evol. 2009;50: 534–546. 10.1016/j.ympev.2008.11.007 19049884

[26] Kotov AA, Ishida S, Taylor DJ. Revision of the genus *Bosmina* Baird, 1845 (Cladocera: Bosminidae), based on evidence from male morphological characters and molecular phylogenies. Zool J Linn Soc. 2009;156: 1–56.

[27] Xu S, Hebert PDN, Kotov AA, Cristescu ME. The non-cosmopolitanism paradigm of freshwater zooplankton: insights from the global phylogeography of the predatory cladoceran *Polyphemus pediculus* (Crustacea, Onychopoda). Mol Ecol. 2009;18: 5161–5179. 10.1111/j.1365-294X.2009.04422.x 19912535

[28] Xu L, Han BP, Van Damme K, Vierstraete A, Vanfleteren JR, Dumont HJ. Biogeography and evolution of the Holarctic zooplankton genus *Leptodora* (Crustacea: Branchiopoda: Haplopoda). J Biogeogr. 2010;38: 359–370.

[29] Faustová M, Sacherová V, Sheets HD, Svensson JE, Taylor DJ. Coexisting cyclic parthenogens comprise a Holocene species flock in *Eubosmina*. PLoS ONE. 2010; 5 (7): e11623 10.1371/journal.pone.0011623 20661283 PMC2905414

[30] Huang X, Shi X, Kotov AA, Gu F. Confirmation through genetic analysis of the existence of many local phyloclades of the genus *Simocephalus* (Crustacea, Cladocera) in China. PLoS ONE. 2014;9(11): e112808 10.1371/journal.pone.0112808 25393020 PMC4231159

[31] Hebert PDN, Ratnasingham S, deWaard J. Barcoding animal life: cytochrome c oxidase subunit 1 divergences among closely related species. Proc Biol Sci. 2003;270: S96–S99. 12952648 10.1098/rsbl.2003.0025 PMC1698023

[32] Hebert PDN, Cywinska A, Ball SL, De Waard JR. Biological identifications through DNA barcodes. Proc Biol Sci. 2003;270: 313–321. 12614582 10.1098/rspb.2002.2218 PMC1691236

[33] Costa FO, DeWaard JR, Boutillier J, Ratnasingham S, Dooh RT, Hajibabaei M, et al Biological identifications through DNA barcodes: the case of the Crustacea. Can J Fish Aquat Sci. 1997;64: 272–295.

[34] DeWaard JR, Sacherova V, Cristescu MEA, Remigio EA, Crease TJ, Hebert PDN. Probing the relationships of the branchiopod crustaceans. Mol Phylogenet Evol. 2006;39: 491–502. 16406819 10.1016/j.ympev.2005.11.003

[35] Elías-Gutiérrez M, Martínez Jerónimo F, Ivanova NV, Valdez Moreno M, Hebert PDN. DNA barcodes for Cladocera and Copepoda from Mexico and Guatemala, highlights and new discoveries. Zootaxa. 2008;1839: 1–42.

[36] Jeffery NW, Elías-Gutiérrez M, Adamowicz SJ. Species diversity and phylogeographical affinities of the Branchiopoda (Crustacea) of Churchill, Manitoba, Canada. PLoS ONE. 2011;6(5): e18364 10.1371/journal.pone.0018364 21610864 PMC3096620

[37] Elías-Gutiérrez M, Valdez-Moreno M. A new cryptic species of *Leberis* Smirnov, 1989 (Crustacea, Cladocera, Chydoridae) from the Mexican semi-desert region, highlighted by DNA barcoding. Hidrobiologica. 2008;18: 63–74.

[38] Quiroz-Vázquez P, Elías-Gutiérrez M. A new cryptic species of the freshwater cladoceran genus Scapholeberis Schoedler, 1858 (Cladocera: Anomopoda) from the Semidesert Northern Mexico, highlighted by DNA barcoding. Zootaxa. 2009;2236: 50–64.

[39] Kotov AA, Taylor DJ. A new African lineage of the *Daphnia obtusa* group (Cladocera: Daphniidae) disrupts continental vicariance patterns. J Plankton Res. 2010;32: 937–949.

[40] Bekker EI, Kotov AA, Taylor DJ. A revision of the subgenus *Eurycercus* (*Eurycercus*) Baird, 1843 emend. nov. (Cladocera: Eurycercidae) in the Holarctic with the description of a new species from Alaska. Zootaxa. 2012;3206: 1–40.

[41] Eitam A, Blaustein L, Van Damme K, Dumont HJ, Martens K. Crustacean species richness in temporary pools: relationships with habitat traits. Hydrobiologia. 2004;525: 125–130.

[42] Nédli J, De Meester L, Major Á, Schwenk K, Szivák I, Forró L. Salinity and depth as structuring factors of cryptic divergence in *Moina brachiata* (Crustacea: Cladocera). Fundamental Appl Limnol / Arch Hydrobiol. 2014;184: 69–85.

[43] Goulden CE. The systematics and evolution of the Moinidae. Trans Am Philos Soc Philadelp, new series. 1968;58: 1–101.

[44] Smirnov NN. Macrothricidae and Moinidae of the World fauna. Fauna SSSR, Novaya Seriya. 1976;1(3): 1–237 (In Russian).

[45] Wong CK, Chu KH, Shum FF. Acute and chronic toxicity of malathion to the freshwater cladoceran *Moina macrocopa*. Water Air Soil Pollut. 1995;84: 399–405.

[46] Mangas-Ramírez E, Sarma SSS, Nandini S. Combined effects of algal (*Chlorella vulgaris*) density and ammonia concentration on the population dynamics of *Ceriodaphnia dubia* and *Moina macrocopa* (Cladocera). Ecotoxicol Environ Saf. 2002;51: 216–222. 11971644 10.1006/eesa.2001.2128

[47] Smirnov NN. Physiology of the Cladocera. London etc.: Academic Press; 2014.

[48] Alonso M. Crustacea, Branchiopoda. Fauna Iberica 7. Crustacea Branchiopoda. Madrid: Museo Nacional de Ciencias Naturales, Consejo Superior de Investigaciones Científicas; 1996.

[49] Forró L. A new species of *Moina* from Australia (Crustacea: Cladocera). Acta Zool Hung. 1985;31: 111–118.

[50] Forró L. *Moina kaszabi* sp.n. (Crustacea, Cladocera), its separation from M. belli Gurney by multivariative analyses. Acta Zool Hung. 1988;34; 203–214.

[51] Forró L. *Moina salina* Daday, 1888 (Cladocera, Moinidae)–a new species for the Bulgarian fauna. Acta Zool Bulgar. 1990;39: 78–80.

[52] Forró L. Mating behaviour in *Moina brachiata* (Jurine, 1820) (Crustacea, Anomopoda). Hydrobiologia. 1997;360: 153–159.

[53] Hudec I. Occurrence and biology of *Moina micrura* Kurz 1874 and *Moina weismanni* Ishikawa 1896 (Crustacea, Cladocera) in Slovakia. Biologia. 1988;43: 871–881.

[54] Hudec I. *Moina weismanni* Ishikawa, 1896 (Cladocera, Moinidae) in Central Europe. Hydrobiologia. 1990;190: 33–42.

[55] Hudec I. *Moina ephemeralis* n.sp. from Central Europe. Hydrobiologia. 1997;360: 55–61.

[56] Hudec I. Anomopoda, Ctenopoda, Haplopoda, Onychopoda (Crustacea: Branchiopoda). Fauna Slovenska III. Bratislava: VEDA; 2010.

[57] Mirabdullaev IM. *Moina weismanni* (Crustacea, Cladocera): a species new for Russia and central Asia. Zool Zhurnal. 1992;71: 136–139.

[58] Mirabdullaev IM. *Moina gouldeni* n.sp. (Cladocera, Moinidae) from Central Asia. Crustaceana. 1993;64: 192–196.

[59] Mirabdullaev IM. *Moina mukhamedievi* n. sp. (Crustacea, Cladocera) from ricefields of Uzbekistan (central Asia). Hydrobiologia. 1998;385: 11–16.

[60] Margaritora FG, Ferrari I, Crosetti D. A Far East *Moina*, *M*. *weismanni* Ishikawa, 1896 found in an Italian ricefield. Hydrobiologia. 1987;145: 93–103.

[61] Martínez-Jerónimo F, Elías-Gutiérrez M, Suárez-Morales E. A redescription of *Moina hutchinsoni*, a rare cladoceran (Branchiopoda: Anomopoda) found in remnants of a Mexican saline lake, with notes on its life history. J Crustacean Biol. 2004;24: 232–245.

[62] Kotov AA, Elías-Gutiérrez M, Granados-Ramírez JG. *Moina dumonti* sp. nov. (Cladocera, Anomopoda, Moinidae) from Southern Mexico and Cuba, with comments on moinid limbs. Crustaceana. 2005;78: 41–57.

[63] Paggi JC. *Moina macrocopa* (Straus, 1820) (Branchiopoda, Anomopoda) in South America: Another case of species introduction? Crustaceana. 1997;70: 886–893.

[64] Van Damme K, Dumont HJ. A new species of *Moina* Baird, 1950 (Crustacea: Anomopoda) from Socotra Island, Yemen. Zootaxa. 2008;1721: 24–34.

[65] Dumont HJ, Rietzler AC, Kalapothakis E. *Micromoina arboricola* n. gen., n. spec. (Crustacea: Cladocera), a new moinid living in a forest tree-hole in Minas Gerais, Brazil. Zootaxa. 2013;3652: 533–546. 26269853 10.11646/zootaxa.3652.5.3

[66] Padhye SM, Dumont HJ. *Moina hemanti* sp. nov., a new species of the genus *Moina* s.l. (Branchiopoda: Anomopoda) from Pune, India. Zootaxa. 2014;3860: 561–570. 10.11646/zootaxa.3860.6.4 25283291

[67] Petrusek A, Černy M, Audenaert E. Large intercontinental differentiation of *Moina micrura* (Crustacea: Anomopoda): one less cosmopolitan cladoceran? Hydrobiologia. 2004;526: 73–81.

[68] Chatterjee T, Kotov AA, Van Damme K, Chandrasekhar SVA, Padhye S. An annotated checklist of the Cladocera (Crustacea: Branchiopoda) from India. Zootaxa. 2013;3667: 1–89. 26079003 10.11646/zootaxa.3667.1.1

[69] Tatsuta H, Yao I, Tanaka Y. Isolation of eight microsatellite markers from *Moina macrocopa* for assessing cryptic genetic structure in the wild. Mol Ecol Resour. 2009;9: 904–906. 10.1111/j.1755-0998.2008.02410.x 21564785

[70] Prosser S, Martínez-Arce A, Elías-Gutiérrez M. A new set of primers for COI amplification from freshwater microcrustaceans. Mol Ecol Resour. 2013;13: 1151–1155. 10.1111/1755-0998.12132 23795700

[71] Kotov AA, Forró L, Korovchinsky NM, Petrusek A. World checklist of freshwater Cladocera species. World Wide Web electronic publication. 2013. Available: http://fada.biodiversity.be/group/show/17

[72] Smirnov NN. Family Moinidae. In: Alekseev VR, editor. Key to freshwater invertebrates of Russia, Vol. 2 S.- Petersburg: Zoological Institute Press; 1995 pp. 64–66.

[73] Geller J, Meyer C, Parker M, Hawk H. Redesign of PCR primers for mitochondrial cytochrome *c* oxidase subunit I for marine invertebrates and application in all-taxa biotic surveys. Mol Ecol Resour. 2013;13: 851–861. 10.1111/1755-0998.12138 23848937

[74] Messing J. New M13 vectors for cloning. Method Enzymol. 1983;101: 29–71. 10.1016/0076-6879(83)01005-8 6310323

[75] Linhart C, Shamir R. The degenerate primer design problem: theory and applications. J Comput Biol. 2005;12: 431–456. 15882141 10.1089/cmb.2005.12.431

[76] Regier JC, Shi D. 2005. Increased yield of PCR product from degenerate primers with nondegenerate, nonhomologous 5' tails. Biotechniques. 2005;38: 34–38. 15679081 10.2144/05381BM02

[77] Okonechnikov K, Golosova O, Fursov M. Unipro UGENE: a unified bioinformatics toolkit. Bioinformatics. 2012;28: 1166–1167. 10.1093/bioinformatics/bts091 22368248

[78] Larkin M, Blackshields G, Brown N, Chenna R, McGettigan PA, McWilliam H, et al ClustalW and ClustalX version 2. Bioinformatics. 2007;23: 2947–2948. 17846036 10.1093/bioinformatics/btm404

[79] Librado P, Rozas J. DnaSP v5: a software for comprehensive analysis of DNA polymorphism data. Bioinformatics. 2009;25: 1451–1452. 10.1093/bioinformatics/btp187 19346325

[80] Tamura K, Stecher G, Peterson D, Filipski A, Kumar S. MEGA6: Molecular Evolutionary Genetics Analysis Version 6.0. Mol Biol Evol. 2013;30: 2725–2729 10.1093/molbev/mst197 24132122 PMC3840312

[81] Darriba D, Taboada G, Doallo R, Posada D. jModelTest 2: more models, new heuristics and parallel computing. Nature Meth. 2012;9: 772. 10.1038/nmeth.2109 PMC4594756 22847109

[82] Posada D, Buckley T. Model selection and model averaging in phylogenetics: advantages of Akaike Information Criterion and Bayesian approaches over likelihood ratio tests. Syst Biol. 2004;53: 793–808. 15545256 10.1080/10635150490522304

[83] Rodriguez F, Oliver J, Marin A, Medina J. The general stochastic model of nucleotide substitution. J Theor Biol. 1990;142: 485–501. 2338834 10.1016/s0022-5193(05)80104-3

[84] Ronquist F, Teslenko M, van der Mark P., et al MrBayes 3.2: Efficient Bayesian phylogenetic inference and model choice across a large model space. Syst Biol. 2012;6: 539–542. 10.1093/sysbio/sys029 PMC3329765 22357727

[85] Tamura K. Estimation of the number of nucleotide substitutions when there are strong transition-transversion and G+C-content biases. Mol Biol Evol. 1992;9: 678–687. 1630306 10.1093/oxfordjournals.molbev.a040752

[86] Wang W, Zhang K, Deng D, Zhang Y-N, Peng S, Xu X. Genetic diversity of *Daphnia pulex* in the Middle and Lower Reaches of the Yangtze River. PLoS ONE. 2016;11(3): e0152436 10.1371/journal.pone.0152436 27015539 PMC4807850

[87] Dudov KP, Perry RP. The gene family encoding the mouse ribosomal protein L32 contains a uniquely expressed intron-containing gene and an unmutated processed gene. Cell. 1984;37: 457–468. 6327068 10.1016/0092-8674(84)90376-3

[88] Vanin EF. Processed pseudogenes: characteristics and evolution. Annu Rev Genet. 1985;19: 253–72. 3909943 10.1146/annurev.ge.19.120185.001345

[89] Song H, Buhay JE, Whiting MF, Crandall KA. Many species in one: DNA barcoding overestimates the number of species when nuclear mitochondrial pseudogenes are coamplified. Proc Natl Acad Sci USA. 2008,105: 13486–13491. 10.1073/pnas.0803076105 18757756 PMC2527351

[90] Buhay JE. “*COI*-like” sequences are becoming problematic in molecular systematic and DNA barcoding studies. J Crustacean Biol. 2009;29: 96–110.

[91] Adamowicz SJ, Purvis A. How many branchiopod crustacean species are there? Quantifying the components of underestimation. Glob Ecol Biogeogr. 2005;14: 455–468.

[92] Smirnov NN, Timms BV. A revision of the Australian Cladocera (Crustacea). Rec Austal Mus Suppl. 1983;1: 1–132.

[93] Petrusek A, Thielsch A, Schwenk K. Mitochondrial sequence variation suggests extensive cryptic diversity within the Western Palearctic *Daphnia longispina* complex. Limnol Oceanogr. 2012;57: 1838–1845.

[94] Van Damme K, Kotov AA, Dumont HJ. A checklist of names in Alona Baird 1843 (Crustacea: Cladocera: Chydoridae) and their current status: an analysis of the taxonomy of a lump genus. Zootaxa. 2010;2330: 1–63.

[95] Elías-Gutiérrez M, Zamuriano-Claros R. Primer registro de *Moina macrocopa* (Daphniiformes: Moinidae) en Bolivia. Rev Biol Trop. 1994;42: 385.

[96] Elmoor-Loureiro LMA, Santangelo JM, Lopes PM, Bozelli RL. A new report of *Moina macrocopa* (Straus, 1820) (Cladocera, Anomopoda) in South America. Braz J Biol. 2010;70: 225–226. 20231982 10.1590/s1519-69842010000100031

[97] Vignatti AM, Cabrera GC, Echaniz SA. Distribution and biological aspects of the introduced species *Moina macrocopa* (Straus, 1820) (Crustacea, Cladocera) in the semi-arid central region of Argentina. Biota Neotrop. 2013;13: 86–92.

[98] Kotov AA, Taylor DJ. Mesozoic fossils (>145 Mya) suggest the antiquity of the subgenera of *Daphnia* and their coevolution with chaoborid predators. BMC Evol Biol. 2011;11: 129 10.1186/1471-2148-11-129 21595889 PMC3123605

[99] Smirnov NN. Mesozoic Anomopoda (Crustacea) from Mongolia. Zool J Linn Soc. 1992;104: 97–116.

[100] Schwenk K. Interspecific hybridization in *Daphnia*: distinction and origin of hybrid matrilines. Molec Biol Evol. 1993;10: 1289–1302. 8277855 10.1093/oxfordjournals.molbev.a040076

[101] Schwenk K, Posada D., Hebert PDN. Molecular systematics of European *Hyalodaphnia*: the role of contemporary hybridization in ancient species. Proc Biol Sci. 2000;267: 1833–1842. 11052533 10.1098/rspb.2000.1218 PMC1690753

[102] Billiones R, Brehm M, Klee J, Schwenk K. Genetic identification of *Hyalodaphnia* species and interspecific hybrids. Hydrobiologia. 2004;526: 43–53.

[103] Ishida S, Taylor DJ. Quaternary diversification in a sexual Holarctic zooplankter, *Daphnia galeata*. Mol Ecol. 2007;16: 569–582. 17257114 10.1111/j.1365-294X.2006.03160.x

[104] Dlouhá S, Thielsch A, Kraus RHS, Seda J, Schwenk K, Petrusek A. Identifying hybridizing taxa within the *Daphnia longispina* species complex: which methods to rely on? Hydrobiologia. 2010;643: 107–122.

[105] Ishida S, Takahashi A, Matsushima N, Yokoyama J, Makino W, Urabe J, et al 2011. The long-term consequences of hybridization between the two *Daphnia* species, *D*. *galeata* and *D*. *dentifera*, in mature habitats. BMC Evol Biol. 2011;11: 209 10.1186/1471-2148-11-209 21756366 PMC3156774

[106] International Commission on Zoological Nomenclature. International code of zoological nomenclature. Fourth edition London: The Natural History Museum; 2000.

[107] Kotov AA. A critical review of the current taxonomy of the genus *Daphnia* O. F. Müller, 1785. Zootaxa. 2015;3911: 184–200. 10.11646/zootaxa.3911.2.2 25661605

[108] Quicke DLJ. Principles and techniques of contemporary taxonomy. London etc: Blackie Academic & Professional Press; 1993.

[109] Behning AL. Das Leben der Volga. Die Binnengewasser. 1928;5: 1–162.

[110] Yevdokimov NA, Yermokhin MV. Crustaceans of zooplankton in temporary waterbodies of Saratov District on territory of different natural zones. Biol Vnutrennikh Vod. 2009;1: 62–69.

[111] Burak ES. Life tables of *Moina macrocopa* (Straus) in successive generations under food and temperature adaptation. Hydrobiologia. 1997;360: 101–108.

[112] Benider A, Tifnouti A, Pourriot R. Growth of *Moina macrocopa* (Straus 1820) (Crustacea, Cladocera): influence of trophic conditions, population density and temperature. Hydrobiologia. 2002;468: 1–11.

[113] Flössner D. Krebstiere, Crustacea (Kiemen- und Blattfüßer, Branchiopoda, Fischläuse, Branchiura). Die Tierwelt Deutschlands. 1972;60: 1–499.

[114] Flössner D. Die Haplopoda und Cladocera (ohne Bosminidae) Mitteleuropas. Leiden: Backhuys; 2000.

