# Supplementary material for: DNA Barcoding Reveals High Cryptic Diversity in the North Eurasian Moina Species (Crustacea: Cladocera)
Source: PLoS One. 2016 Aug 24;11(8):e0161737. doi: 10.1371/journal.pone.0161737 (PMC4996527; doi:10.1371/journal.pone.0161737)
Supplement: S1 Table — Clade designations correspond to those in other tables. AR in list of states = Autonomous Republic. (DOC) [file pone.0161737.s001.doc]

**S1 Table.** Complete list of sequences obtained in this study with information on locality, haplotype number and the GenBank accession number provided for each specimen. Clade designations correspond to those in other tables. AR in list of states = Autonomous Republic.

| **Phylogroup codes** | **Phylogroup name** | **Sequence unique ID** | **Country of origin** | **State** | **Locality** | **Latitude, °N** | **Longitude, °E** | **GenBank accession numbers** | **Haplotype** |
| --- | --- | --- | --- | --- | --- | --- | --- | --- | --- |
| 1 | *Moina* cf. *brachiata* clade A | Zhm_280 | Russia (European) | Rostov Area | A puddle in steppe | 46.43333 | 42.73333 | KX168584 | H_58 |
| 1 | *Moina* cf. *brachiata* clade A | Zhm_104 | Russia (European) | Rostov Area | Lopukhovatoe Lake | 46.46667 | 42.800687 | KX168530 | H_25 |
| 1 | *Moina* cf. *brachiata* clade A | Zhm_221 | Russia (European) | Samara Area | Unnamed pond | 52.09518 | 50.85844 | KX168577 | H_54 |
| 1 | *Moina* cf. *brachiata* clade A | Zhm_222 | Russia (European) | Samara Area | Unnamed pond | 52.09518 | 50.85844 | KX168577 | H_54 |
| 1 | *Moina* cf. *brachiata* clade A | Zhm_223 | Russia (European) | Samara Area | Unnamed pond | 52.09518 | 50.85844 | KX168577 | H_54 |
| 1 | *Moina* cf. *brachiata* clade A | Zhm_103 | Russia (Asian) | Novosibirsk Area | A pond, Novofeklino | 55.12771 | 77.03781 | KX168529 | H_24 |
| 1 | *Moina* cf. *brachiata* clade A | Zhm_127 | Russia (Asian) | Novosibirsk Area | pond in a village | 55.12783 | 77.03733 | KX168542 | H_24 |
| 1 | *Moina* cf. *brachiata* clade A | Zhm_128 | Russia (Asian) | Novosibirsk Area | pond in a village | 55.12783 | 77.03733 | KX168543 | H_36 |
| 1 | *Moina* cf. *brachiata* clade A | Zhm_130 | Russia (Asian) | Novosibirsk Area | pond in a village | 55.12783 | 77.03733 | KX168543 | H_36 |
| 1 | *Moina* cf. *brachiata* clade A | Zhm_195 | Russia (Asian) | Novosibirsk Area | Suzdalka lake | 54.577 | 79.45766 | KX168569 | H_48 |
| 1 | *Moina* cf. *brachiata* clade A | Zhm_196 | Russia (Asian) | Novosibirsk Area | Suzdalka lake | 54.577 | 79.45766 | KX168569 | H_48 |
| 1 | *Moina* cf. *brachiata* clade A | Zhm_197 | Russia (Asian) | Novosibirsk Area | Suzdalka lake | 54.577 | 79.45766 | KX168569 | H_48 |
| 1 | *Moina* cf. *brachiata* clade A | Zhm_198 | Russia (Asian) | Novosibirsk Area | Suzdalka lake | 54.577 | 79.45766 | KX168569 | H_48 |
| 1 | *Moina* cf. *brachiata* clade A | Zhm_205 | Russia (Asian) | Tyumen Area | small puddle near a farm | 55.71367 | 68.991 | KX168572 | H_49 |
| 1 | *Moina* cf. *brachiata* clade A | Zhm_209 | Russia (Asian) | Tyumen Area | watering pond near the road | 55.77567 | 68.07767 | KX168572 | H_49 |
| 1 | *Moina* cf. *brachiata* clade A | Zhm_210 | Russia (Asian) | Tyumen Area | watering pond near the road | 55.77567 | 68.07767 | KX168572 | H_49 |
| 1 | *Moina* cf. *brachiata* clade A | Zhm_213 | Kazakhstan |  | A small pudle near Ural River | 47.21351 | 51.95353 | KX168573 | H_50 |
| 1 | *Moina* cf. *brachiata* clade A | Zhm_215 | Kazakhstan |  | A small pudle near Ural River | 47.21351 | 51.95353 | KX168574 | H_51 |
| 1 | *Moina* cf. *brachiata* clade A | Zhm_216 | Kazakhstan |  | A small pudle near Ural River | 47.21351 | 51.95353 | KX168575 | H_52 |
| 2 | *Moina* cf. *brachiata* clade B | Zhm_199 | Russia (Asian) | Altai Territory | Unnamed lake | 49.97417 | 88.68816 | KX168570 | H_15 |
| 2 | *Moina* cf. *brachiata* clade B | Zhm_200 | Russia (Asian) | Altai Territory | Unnamed lake | 49.97417 | 88.68816 | KX168570 | H_15 |
| 2 | *Moina* cf. *brachiata* clade B | Zhm_201 | Russia (Asian) | Altai Territory | Unnamed lake | 49.97417 | 88.68816 | KX168570 | H_15 |
| 2 | *Moina* cf. *brachiata* clade B | Zhm_202 | Russia (Asian) | Altai Territory | Unnamed lake | 49.97417 | 88.68816 | KX168570 | H_15 |
| 2 | *Moina* cf. *brachiata* clade B | Zhm_078 | Russia (Asian) | Altai Territory | A pond, Kosh-Agach | 49.99181 | 88.66839 | KX168520 | H_15 |
| 2 | *Moina* cf. *brachiata* clade B | Zhm_079 | Russia (Asian) | Altai Territory | A pond, Kosh-Agach | 49.99181 | 88.66839 | KX168521 | H_16 |
| 2 | *Moina* cf. *brachiata* clade B | Zhm_080 | Russia (Asian) | Altai Territory | A pond, Kosh-Agach | 49.99181 | 88.66839 | KX168521 | H_16 |
| 2 | *Moina* cf. *brachiata* clade B | Zhm_081 | Russia (Asian) | Altai Territory | A pond, Kosh-Agach | 49.99181 | 88.66839 | KX168520 | H_15 |
| 2 | *Moina* cf. *brachiata* clade B | Zhm_138 | Russia (Asian) | Tuva AR | Haka-Hol salt lake | 51.33117 | 93.57333 | KX168547 | H_38 |
| 2 | *Moina* cf. *brachiata* clade B | Zhm_139 | Russia (Asian) | Tuva AR | Haka-Hol salt lake | 51.33117 | 93.57333 | KX168548 | H_16 |
| 2 | *Moina* cf. *brachiata* clade B | Zhm_140 | Russia (Asian) | Tuva AR | Haka-Hol salt lake | 51.33117 | 93.57333 | KX168548 | H_16 |
| 2 | *Moina* cf. *brachiata* clade B | Zhm_141 | Russia (Asian) | Tuva AR | Haka-Hol salt lake | 51.33117 | 93.57333 | KX168547 | H_38 |
| 2 | *Moina* cf. *brachiata* clade B | Zhm_001 | Russia (Asian) | Irkutsk Area | Shara-Nur Lake, Olkhon Island, Baikal | 53.104535 | 107.255493 | KX168502 | H_01 |
| 2 | *Moina* cf. *brachiata* clade B | Zhm_002 | Russia (Asian) | Irkutsk Area | Shara-Nur Lake | 53.104535 | 107.255493 | KX168503 | H_01 |
| 2 | *Moina* cf. *brachiata* clade B | Zhm_168 | China |  | Namtso Chukmo Lake | 30.748 | 90.871 | KX168557 | H_16 |
| 2 | *Moina* cf. *brachiata* clade B | Zhm_169 | China |  | Namtso Chukmo Lake | 30.748 | 90.871 | KX168557 | H_16 |
| 2 | *Moina* cf. *brachiata* clade B | Zhm_170 | China |  | Namtso Chukmo Lake | 30.748 | 90.871 | KX168557 | H_16 |
| 2 | *Moina* cf. *brachiata* clade B | Zhm_171 | Mongolia |  | Unnamed puddle | 47.0808 | 105.955 | KX168558 | H_16 |
| 2 | *Moina* cf. *brachiata* clade B | Zhm_172 | Mongolia |  | Unnamed puddle | 47.0808 | 105.955 | KX168558 | H_16 |
| 2 | *Moina* cf. *brachiata* clade B | Zhm_173 | Mongolia |  | Unnamed puddle | 47.0808 | 105.955 | KX168558 | H_16 |
| 2 | *Moina* cf. *brachiata* clade B | Zhm_010 | Mongolia |  | A puddle near Oygon Nuur | 47.94528 | 93.24277 | KX168508 | H_06 |
| 2 | *Moina* cf. *brachiata* clade B | Zhm_011 | Mongolia |  | A puddle near Oygon Nuur | 47.94528 | 93.24277 | KX168508 | H_06 |
| 2 | *Moina* cf. *brachiata* clade B | Zhm_013 | Mongolia |  | A puddle near Oygon Nuur | 47.94528 | 93.24277 | KX168508 | H_06 |
| 3 | *Moina* cf. brachiata clade C | Zhm_004 | Russia (European) | Saratov Area | A pond near oxbow lake, Verkhny Yeruslan | 50.50928 | 46.51882 | KX168505 | H_03 |
| 3 | *Moina* cf. brachiata clade C | Zhm_005 | Russia (European) | Saratov Area | A pond near oxbow lake, Verkhny Yeruslan | 50.50928 | 46.51882 | KX168506 | H_04 |
| 3 | *Moina* cf. brachiata clade C | Zhm_183 | Russia (European) | Saratov Area | A puddle, Piterka village | 50.61300 | 47.44808 | KX168563 | H_3 |
| 3 | *Moina* cf. brachiata clade C | Zhm_184 | Russia (European) | Saratov Area | A puddle, Piterka village | 50.61300 | 47.44808 | KX168564 | H_46 |
| 3 | *Moina* cf. brachiata clade C | Zhm_185 | Russia (European) | Saratov Area | A puddle, Piterka village | 50.61300 | 47.44808 | KX168563 | H_3 |
| 3 | *Moina* cf. brachiata clade C | Zhm_186 | Russia (European) | Saratov Area | A puddle, Piterka village | 50.61300 | 47.44808 | KX168563 | H_3 |
| 3 | *Moina* cf. brachiata clade C | Zhm_187 | Russia (European) | Saratov Area | A puddle, Verkhny Yeruslan | 50.51442 | 46.52524 | KX168565 | H_4 |
| 3 | *Moina* cf. brachiata clade C | Zhm_188 | Russia (European) | Saratov Area | A puddle, Verkhny Yeruslan | 50.51442 | 46.52524 | KX168566 | H_3 |
| 3 | *Moina* cf. brachiata clade C | Zhm_189 | Russia (European) | Saratov Area | A puddle, Verkhny Yeruslan | 50.51442 | 46.52524 | KX168566 | H_3 |
| 3 | *Moina* cf. brachiata clade C | Zhm_190 | Russia (European) | Saratov Area | A puddle, Verkhny Yeruslan | 50.51442 | 46.52524 | KX168566 | H_3 |
| 4 | *Moina* cf. *brachiata* clade D | Zhm_083 | Ukraine | Odessa Area | Cow pond near River Tiligul | 47.1657 | 30.9269 | KX168522 | H_17 |
| 4 | *Moina* cf. *brachiata* clade D | Zhm_085 | Ukraine | Odessa Area | Cow pond near River Tiligul | 47.1657 | 30.9269 | KX168522 | H_17 |
| 4 | *Moina* cf. *brachiata* clade D | Zhm_175 | Russia (European) | Saratov Area | A puddle, Novozakharkino village | 52.19113 | 45.32003 | KX168559 | H_17 |
| 4 | *Moina* cf. *brachiata* clade D | Zhm_176 | Russia (European) | Saratov Area | A puddle, Novozakharkino village | 52.19113 | 45.32003 | KX168560 | H_44 |
| 4 | *Moina* cf. *brachiata* clade D | Zhm_177 | Russia (European) | Saratov Area | A puddle, Novozakharkino village | 52.19113 | 45.32003 | KX168559 | H_17 |
| 4 | *Moina* cf. *brachiata* clade D | Zhm_178 | Russia (European) | Saratov Area | A puddle, Novozakharkino village | 52.19113 | 45.32003 | KX168561 | H_39 |
| 4 | *Moina* cf. *brachiata* clade D | Zhm_142 | Russia (European) | Bashkortostan AR | geese farm pouddle | 54.7285 | 55.18067 | KX168549 | H_39 |
| 4 | *Moina* cf. *brachiata* clade D | Zhm_144 | Russia (European) | Bashkortostan AR | geese farm pouddle | 54.7285 | 55.18067 | KX168551 | H_41 |
| 4 | *Moina* cf. *brachiata* clade D | Zhm_145 | Russia (European) | Bashkortostan AR | geese farm pouddle | 54.7285 | 55.18067 | KX168549 | H_39 |
| 4 | *Moina* cf. *brachiata* clade D | Zhm_146 | Russia (European) | Bashkortostan AR | geese farm pouddle | 54.7285 | 55.18067 | KX168551 | H_41 |
| 4 | *Moina* cf. *brachiata* clade D | Zhm_102 | Russia (Asian) | Novosibirsk Area | A pond, Novofeklino | 55.12771 | 77.03781 | KX168528 | H_23 |
| 4 | *Moina* cf. *brachiata* clade D | Zhm_129 | Russia (Asian) | Novosibirsk Area | pond in a village | 55.12783 | 77.03733 | KX168544 | H_23 |
| 4 | *Moina* cf. *brachiata* clade D | Zhm_131 | Russia (Asian) | Novosibirsk Area | pond in a village | 55.12783 | 77.03733 | KX168544 | H_23 |
| 6 | *Moina* cf. *brachiata* clade F | Zhm_043 | Russia (European) | Saratov Area | Peet lake near Diakovka | 50.6871 | 46.6881 | KX168511 | H_09 |
| 6 | *Moina* cf. *brachiata* clade F | Zhm_044 | Russia (European) | Saratov Area | Peet lake near Diakovka | 50.6871 | 46.6881 | KX168511 | H_09 |
| 6 | *Moina* cf. *brachiata* clade F | Zhm_049 | Russia (European) | Saratov Area | Peet lake near Diakovka | 50.6871 | 46.6881 | KX168513 | H_09 |
| 7 | *Moina* cf. *brachiata* clade G | Zhm_288 | Russia (Asian) | Zabaikalsky Territory | A puddle, Lake Zun-Torey | 50.2167 | 115.63333 | KX168586 | H_27 |
| 7 | *Moina* cf. *brachiata* clade G | Zhm_111 | Russia (Asian) | Zabaikalsky Territory | Bayan-Tsagan lake | 50.32433 | 115.0997 | KX168531 | H_26 |
| 7 | *Moina* cf. *brachiata* clade G | Zhm_112 | Russia (Asian) | Zabaikalsky Territory | Bayan-Tsagan lake | 50.32433 | 115.0997 | KX168532 | H_27 |
| 7 | *Moina* cf. *brachiata* clade G | Zhm_113 | Russia (Asian) | Zabaikalsky Territory | Bayan-Tsagan lake | 50.32433 | 115.0997 | KX168533 | H_28 |
| 7 | *Moina* cf. *brachiata* clade G | Zhm_114 | Russia (Asian) | Zabaikalsky Territory | Bayan-Tsagan lake | 50.32433 | 115.0997 | KX168533 | H_28 |
| 7 | *Moina* cf. *brachiata* clade G | Zhm_191 | Mongolia |  | Taatsin Tsagaan Nuur | 45.14867 | 101.43633 | KX168567 | H_28 |
| 7 | *Moina* cf. *brachiata* clade G | Zhm_192 | Mongolia |  | Taatsin Tsagaan Nuur | 45.14867 | 101.43633 | KX168567 | H_28 |
| 7 | *Moina* cf. *brachiata* clade G | Zhm_193 | Mongolia |  | Taatsin Tsagaan Nuur | 45.14867 | 101.43633 | KX168568 | H_47 |
| 7 | *Moina* cf. *brachiata* clade G | Zhm_194 | Mongolia |  | Taatsin Tsagaan Nuur | 45.14867 | 101.43633 | KX168567 | H_28 |
| 8 | *Moina* cf. *micrura* clade H | Zhm_003 | Russia (European) | Volgograd Area | A pond at border of Saratov and Volgograd Areas | 50.49685 | 47.20553 | KX168504 | H_02 |
| 8 | *Moina* cf. *micrura* clade H | Zhm_042 | Russia (European) | Saratov Area | Peet lake near Diakovka | 50.6871 | 46.6881 | KX168510 | H_08 |
| 8 | *Moina* cf. *micrura* clade H | Zhm_045 | Russia (European) | Saratov Area | Peet lake near Diakovka | 50.6871 | 46.6881 | KX168510 | H_08 |
| 8 | *Moina* cf. *micrura* clade H | Zhm_046 | Russia (European) | Saratov Area | Peet lake near Diakovka | 50.6871 | 46.6881 | KX168512 | H_08 |
| 8 | *Moina* cf. *micrura* clade H | Zhm_047 | Russia (European) | Saratov Area | Peet lake near Diakovka | 50.6871 | 46.6881 | KX168512 | H_08 |
| 8 | *Moina* cf. *micrura* clade H | Zhm_220 | Russia (European) | Samara Area | Unnamed pond | 52.08267 | 50.87328 | KX168576 | H_53 |
| 9 | *Moina* cf. *micrura* clade I | Zhm_100 | Hungary |  | Fülöpszállás, Kelemenszék (Kiskunság National Park) | 46.79338 | 19.17473 | KX168527 | H_22 |
| 9 | *Moina* cf. *micrura* clade I | Zhm_101 | Hungary |  | Fülöpszállás, Kelemenszék (Kiskunság National Park) | 46.79338 | 19.17473 | KX168527 | H_22 |
| 9 | *Moina* cf. *micrura* clade I | Zhm_151 | Russia (European) | Penza Area | A pond in Ramzay village | 53.301 | 44.712 | KX168554 | H_43 |
| 9 | *Moina* cf. *micrura* clade I | Zhm_152 | Russia (European) | Penza Area | A pond in Ramzay village | 53.301 | 44.712 | KX168554 | H_43 |
| 9 | *Moina* cf. *micrura* clade I | Zhm_153 | Russia (European) | Penza Area | A pond in Ramzay village | 53.301 | 44.712 | KX168555 | H_22 |
| 9 | *Moina* cf. *micrura* clade I | Zhm_154 | Russia (European) | Penza Area | A pond in Ramzay village | 53.301 | 44.712 | KX168555 | H_22 |
| 9 | *Moina* cf. *micrura* clade I | Zhm_232 | Kazakhstan |  | Unnamed channel | 46.62181 | 49.52379 | KX168579 | H_43 |
| 10 | *Moina* cf. *micrura* clade J | Zhm_235 | Kazakhstan |  | Unnamed water reservoir | 49.22848 | 50.69797 | KX168580 | H_56 |
| 10 | *Moina* cf. *micrura* clade J | Zhm_236 | Kazakhstan |  | Unnamed water reservoir | 49.22848 | 50.69797 | KX168580 | H_56 |
| 16 | *Moina* *lipini* | Zhm_094 | Russia (European) | Moscow Area | A fishpond, Yakot | 56.42484 | 37.60246 | KX168525 | H_20 |
| 16 | *Moina* *lipini* | Zhm_095 | Russia (European) | Moscow Area | A fishpond, Yakot | 56.42484 | 37.60246 | KX168526 | H_21 |
| 16 | *Moina* *lipini* | Zhm_096 | Russia (European) | Moscow Area | A fishpond, Yakot | 56.42484 | 37.60246 | KX168526 | H_21 |
| 16 | *Moina* *lipini* | Zhm_097 | Russia (European) | Moscow Area | A fishpond, Yakot | 56.42484 | 37.60246 | KX168526 | H_21 |
| 16 | *Moina* *lipini* | Zhm_179 | Russia (European) | Saratov Area | A small pond near a farm | 50.49265 | 47.36522 | KX168562 | H_45 |
| 16 | *Moina* *lipini* | Zhm_181 | Russia (European) | Saratov Area | A small pond near a farm | 50.49265 | 47.36522 | KX168562 | H_45 |
| 16 | *Moina* *lipini* | Zhm_182 | Russia (European) | Saratov Area | A small pond near a farm | 50.49265 | 47.36522 | KX168562 | H_45 |
| 18 | *Moina* cf. *macrocopa* clade L | Zhm_119 | Russia (Asian) | Zabaikalsky Territory | Zun-Torey lake | 50.22583 | 115.642 | KX168536 | H_31 |
| 18 | *Moina* cf. *macrocopa* clade L | Zhm_120 | Russia (Asian) | Zabaikalsky Territory | Zun-Torey lake | 50.22583 | 115.642 | KX168536 | H_31 |
| 18 | *Moina* cf. *macrocopa* clade L | Zhm_121 | Russia (Asian) | Zabaikalsky Territory | Zun-Torey lake | 50.22583 | 115.642 | KX168536 | H_31 |
| 18 | *Moina* cf. *macrocopa* clade L | Zhm_122 | Russia (Asian) | Zabaikalsky Territory | Zun-Torey lake | 50.22583 | 115.642 | KX168537 | H_32 |
| 18 | *Moina* cf. *macrocopa* clade L | Zhm_287 | Russia (Asian) | Zabaikalsky Territory | A puddle, Lake Zun-Torey | 50.21667 | 115.63333 | KX168585 | H_32 |
| 18 | *Moina* cf. *macrocopa* clade L | Zhm_289 | Russia (Asian) | Zabaikalsky Territory | A puddle, Lake Zun-Torey | 50.21667 | 115.63333 | KX168587 | H_32 |
| 19 | *Moina macrocopa macrocopa* | Zhm_054 | Russia (European) | Karelia AR | A rockpool near Ladoga Lake | 61.47077 | 30.4291 | KX168515 | H_11 |
| 19 | *Moina macrocopa macrocopa* | Zhm_055 | Russia (European) | Karelia AR | A rockpool near Ladoga Lake | 61.47077 | 30.4291 | KX168515 | H_11 |
| 19 | *Moina macrocopa macrocopa* | Zhm_057 | Russia (European) | Karelia AR | A rockpool near Ladoga Lake | 61.47077 | 30.4291 | KX168515 | H_11 |
| 19 | *Moina macrocopa macrocopa* | Zhm_058 | Russia (European) | Karelia AR | A rockpool near Ladoga Lake | 61.47099 | 30.4284 | KX168516 | H_11 |
| 19 | *Moina macrocopa macrocopa* | Zhm_059 | Russia (European) | Karelia AR | A rockpool near Ladoga Lake | 61.47099 | 30.4284 | KX168516 | H_11 |
| 19 | *Moina macrocopa macrocopa* | Zhm_060 | Russia (European) | Karelia AR | A rockpool near Ladoga Lake | 61.47099 | 30.4284 | KX168516 | H_11 |
| 19 | *Moina macrocopa macrocopa* | Zhm_061 | Russia (European) | Karelia AR | A rockpool near Ladoga Lake | 61.47099 | 30.4284 | KX168516 | H_11 |
| 19 | *Moina macrocopa macrocopa* | Zhm_279 | Russia (European) | Rostov Area | A affluent of Lopukhovatoe Lake | 46.45 | 42.78333 | KX168583 | H_57 |
| 19 | *Moina macrocopa macrocopa* | Zhm_143 | Russia (European) | Bashkortostan AR | geese farm pouddle | 54.7285 | 55.18067 | KX168550 | H_40 |
| 19 | *Moina macrocopa macrocopa* | zhm_291 | Russia (Asian) | Tyumen Area | small puddle near a farm | 55.71367 | 68.991 | KX168588 | H_42 |
| 19 | *Moina macrocopa macrocopa* | Zhm_147 | Russia (Asian) | Tyumen Area | small puddle near a farm | 55.71367 | 68.991 | KX168552 | H_42 |
| 19 | *Moina macrocopa macrocopa* | Zhm_150 | Russia (Asian) | Tyumen Area | small puddle near a farm | 55.71367 | 68.991 | KX168553 | H_19 |
| 19 | *Moina macrocopa macrocopa* | Zhm_207 | Russia (Asian) | Tyumen Area | small puddle near a farm | 55.71367 | 68.991 | KX168571 | H_13 |
| 19 | *Moina macrocopa macrocopa* | Zhm_208 | Russia (Asian) | Tyumen Area | small puddle near a farm | 55.71367 | 68.991 | KX168571 | H_13 |
| 19 | *Moina macrocopa macrocopa* | Zhm_066 | Russia (Asian) | Chita Area | Mine lakes near Chernovskaya station | 51.974939 | 113.237256 | KX168518 | H_13 |
| 19 | *Moina macrocopa macrocopa* | Zhm_067 | Russia (Asian) | Chita Area | Mine lakes near Chernovskaya station | 51.974939 | 113.237256 | KX168519 | H_14 |
| 19 | *Moina macrocopa macrocopa* | Zhm_132 | Russia (Asian) | Irkutsk Area | almost dry puddle near Baikal shore | 53.20317 | 107.3503 | KX168545 | H_13 |
| 19 | *Moina macrocopa macrocopa* | Zhm_133 | Russia (Asian) | Irkutsk Area | almost dry puddle near Baikal shore | 53.20317 | 107.3503 | KX168545 | H_13 |
| 19 | *Moina macrocopa macrocopa* | Zhm_134 | Russia (Asian) | Irkutsk Area | almost dry puddle near Baikal shore | 53.20317 | 107.3503 | KX168545 | H_13 |
| 19 | *Moina macrocopa macrocopa* | Zhm_135 | Russia (Asian) | Irkutsk Area | almost dry puddle near Baikal shore | 53.20317 | 107.3503 | KX168546 | H_37 |
| 19 | *Moina macrocopa macrocopa* | Zhm_136 | Russia (Asian) | Irkutsk Area | almost dry puddle near Baikal shore | 53.20317 | 107.3503 | KX168545 | H_13 |
| 19 | *Moina macrocopa macrocopa* | Zhm_092 | Russia (Asian) | Primorski Territory | Bay of Khanka Lake | 44.65 | 132.55 | KX168523 | H_18 |
| 19 | *Moina macrocopa macrocopa* | Zhm_093 | Russia (Asian) | Primorski Territory | Bay of Khanka Lake | 44.65 | 132.55 | KX168524 | H_19 |
| 19 | *Moina macrocopa macrocopa* | Zhm_155 | Russia (Asian) | Primorski Territory | A puddle near Utinoe Lake | 43.37189 | 131.7464 | KX168556 | H_42 |
| 19 | *Moina macrocopa macrocopa* | Zhm_156 | Russia (Asian) | Primorski Territory | A puddle near Utinoe Lake | 43.37189 | 131.7464 | KX168556 | H_42 |
| 19 | *Moina macrocopa macrocopa* | Zhm_157 | Russia (Asian) | Primorski Territory | A puddle near Utinoe Lake | 43.37189 | 131.7464 | KX168556 | H_42 |
| 19 | *Moina macrocopa macrocopa* | Zhm_158 | Russia (Asian) | Primorski Territory | A puddle near Utinoe Lake | 43.37189 | 131.7464 | KX168556 | H_42 |
| 19 | *Moina macrocopa macrocopa* | Zhm_241 | Russia (Asian) | Sakhalin Area | A small muddy puddle near Naiba River | 47.41975 | 142.7794 | KX168581 | H_19 |
| 19 | *Moina macrocopa macrocopa* | Zhm_260 | Russia (Asian) | Kamchatka Area | Puddle in the Avacha River delta | 53.07087 | 158.5363 | KX168582 | H_13 |
| 19 | *Moina macrocopa macrocopa* | Zhm_261 | Russia (Asian) | Kamchatka Area | Puddle in the Avacha River delta | 53.07087 | 158.5363 | KX168582 | H_13 |
| 20 | *Moina* cf. *salina* clade N | Zhm_115 | Russia (Asian) | Zabaikalsky Territory | Tsagan-Nur | 50.37833 | 114.7515 | KX168534 | H_29 |
| 20 | *Moina* cf. *salina* clade N | Zhm_116 | Russia (Asian) | Zabaikalsky Territory | Tsagan-Nur | 50.37833 | 114.7515 | KX168535 | H_30 |
| 20 | *Moina* cf. *salina* clade N | Zhm_117 | Russia (Asian) | Zabaikalsky Territory | Tsagan-Nur | 50.37833 | 114.7515 | KX168534 | H_29 |
| 20 | *Moina* cf. *salina* clade N | Zhm_118 | Russia (Asian) | Zabaikalsky Territory | Tsagan-Nur | 50.37833 | 114.7515 | KX168534 | H_29 |
| 20 | *Moina* cf. *salina* clade N | Zhm_123 | Russia (Asian) | Zabaikalsky Territory | Barun-Torey | 50.16067 | 115.7558 | KX168538 | H_33 |
| 20 | *Moina* cf. *salina* clade N | Zhm_124 | Russia (Asian) | Zabaikalsky Territory | Barun-Torey | 50.16067 | 115.7558 | KX168539 | H_34 |
| 20 | *Moina* cf. *salina* clade N | Zhm_125 | Russia (Asian) | Zabaikalsky Territory | Barun-Torey | 50.16067 | 115.7558 | KX168540 | H_29 |
| 20 | *Moina* cf. *salina* clade N | Zhm_126 | Russia (Asian) | Zabaikalsky Territory | Barun-Torey | 50.16067 | 115.7558 | KX168541 | H_35 |
| 21 | *Moina* cf. *salina* clade O | Zhm_050 | Russia (European) | Rostov Area | Manych-Gudilo Lake | 46.46622 | 42.55372 | KX168514 | H_10 |
| 21 | *Moina* cf. *salina* clade O | Zhm_051 | Russia (European) | Rostov Area | Manych-Gudilo Lake | 46.46622 | 42.55372 | KX168514 | H_10 |
| 21 | *Moina* cf. *salina* clade O | Zhm_052 | Russia (European) | Rostov Area | Manych-Gudilo Lake | 46.46622 | 42.55372 | KX168514 | H_10 |
| 21 | *Moina* cf. *salina* clade O | Zhm_038 | Russia (European) | Astrakhan Area | Gor'kaya River, SW of Bolshaya Bogdo River | 48.22083 | 46.97917 | KX168509 | H_07 |
| 21 | *Moina* cf. *salina* clade O | Zhm_039 | Russia (European) | Astrakhan Area | Gor'kaya River, SW of Bolshaya Bogdo River | 48.22083 | 46.97917 | KX168509 | H_07 |
| 21 | *Moina* cf. *salina* clade O | Zhm_040 | Russia (European) | Astrakhan Area | Gor'kaya River, SW of Bolshaya Bogdo River | 48.22083 | 46.97917 | KX168509 | H_07 |
| 21 | *Moina* cf. *salina* clade O | Zhm_041 | Russia (European) | Astrakhan Area | Gor'kaya River, SW of Bolshaya Bogdo River | 48.22083 | 46.97917 | KX168509 | H_07 |
| 21 | *Moina* cf. *salina* clade O | Zhm_062 | Russia (Asian) | Tuva AR | Lake Khadyn | 51.36427 | 94.50642 | KX168517 | H_12 |
| 21 | *Moina* cf. *salina* clade O | Zhm_226 | Kazakhstan |  | Unnamed pool | 47.19049 | 51.80815 | KX168578 | H_55 |
| 21 | *Moina* cf. *salina* clade O | Zhm_006 | Mongolia |  | Oygon Nuur (lake) | 49.13556 | 96.65833 | KX168507 | H_05 |
| 21 | *Moina* cf. *salina* clade O | Zhm_007 | Mongolia |  | Oygon Nuur (lake) | 49.13556 | 96.65833 | KX168507 | H_05 |
| 21 | *Moina* cf. *salina* clade O | Zhm_008 | Mongolia |  | Oygon Nuur (lake) | 49.13556 | 96.65833 | KX168507 | H_05 |
| 21 | *Moina* cf. *salina* clade O | Zhm_009 | Mongolia |  | Oygon Nuur (lake) | 49.13556 | 96.65833 | KX168507 | H_05 |
