# Supplementary material for: DNA Barcoding Reveals High Cryptic Diversity in the North Eurasian Moina Species (Crustacea: Cladocera)
Source: PLoS One. 2016 Aug 24;11(8):e0161737. doi: 10.1371/journal.pone.0161737 (PMC4996527; doi:10.1371/journal.pone.0161737)
Supplement: S2 Table — (DOC) [file pone.0161737.s002.doc]

**S2 Table.** Complete list of sequences from the GenBank that were used in our study.

| **Phylogroup Number** | **Phylogroup name** | **Sequence unique ID** | **Genbank accession number** | **Country of origin** | **Reference** |
| --- | --- | --- | --- | --- | --- |
| 1 | *Moina* cf. *brachiata* clade A | Moina brachiata Mbra2HU1 | JN641833.1 | Hungary | Nédli et al., 2014 |
| 1 | *Moina* cf. *brachiata* clade A | Moina brachiata Mbra2HU2 | JN641834.1 | Hungary | Nédli et al., 2014 |
| 1 | *Moina* cf. *brachiata* clade A | Moina brachiata MbraHU3 | JN641840.1 | Hungary | Nédli et al., 2014 |
| 1 | *Moina* cf. *brachiata* clade A | Moina brachiata Mbra21HU1 | JN641836.1 | Hungary | Nédli et al., 2014 |
| 1 | *Moina* cf. *brachiata* clade A | Moina brachiata Mbra21HU2 | JN641837.1 | Hungary | Nédli et al., 2014 |
| 1 | *Moina* cf. *brachiata* clade A | Moina brachiata Mbra21HU3 | JN641842.1 | Hungary | Nédli et al., 2014 |
| 1 | *Moina* cf. *brachiata* clade A | Moina brachiata Mbra21HU4 | JN641846.1 | Hungary | Nédli et al., 2014 |
| 1 | *Moina* cf. *brachiata* clade A | Moina brachiata Mbra22HU1 | JN641847.1 | Hungary | Nédli et al., 2014 |
| 1 | *Moina* cf. *brachiata* clade A | Moina brachiata Mbra22HU2 | JN641848.1 | Hungary | Nédli et al., 2014 |
| 1 | *Moina* cf. *brachiata* clade A | Moina brachiata Mbra22HU3 | JN641849.1 | Hungary | Nédli et al., 2014 |
| 2 | *Moina* cf. *brachiata* clade B | Moina rectirostris China | KP027210.1 | China | Chen et al., direct submission |
| 4 | *Moina* cf. *brachiata* clade D | Moina brachiata Mbra4HU | JN641809.1 | Hungary | Nédli et al., 2014 |
| 5 | *Moina* cf. *brachiata* clade E | Moina brachiata Mbra113HU | JN641808.1 | Hungary | Nédli et al., 2014 |
| 5 | *Moina* cf. *brachiata* clade E | Moina brachiata Mbra111HU1 | JN641816.1 | Hungary | Nédli et al., 2014 |
| 5 | *Moina* cf. *brachiata* clade E | Moina brachiata Mbra111HU3 | JN641818.1 | Hungary | Nédli et al., 2014 |
| 5 | *Moina* cf. *brachiata* clade E | Moina brachiata Mbra111HU4 | JN641819.1 | Hungary | Nédli et al., 2014 |
| 5 | *Moina* cf. *brachiata* clade E | Moina brachiata Mbra111HU5 | JN641820.1 | Hungary | Nédli et al., 2014 |
| 5 | *Moina* cf. *brachiata* clade E | Moina brachiata MbraHU1 | JN641823.1 | Hungary | Nédli et al., 2014 |
| 5 | *Moina* cf. *brachiata* clade E | Moina brachiata Mbra112HU | JN641830.1 | Hungary | Nédli et al., 2014 |
| 5 | *Moina* cf. *brachiata* clade E | Moina brachiata Mbra114HU | JN641810.1 | Hungary | Nédli et al., 2014 |
| 5 | *Moina* cf. *brachiata* clade E | Moina brachiata Mbra1HU1 | JN641811.1 | Hungary | Nédli et al., 2014 |
| 5 | *Moina* cf. *brachiata* clade E | Moina brachiata Mbra1HU3 | JN641829.1 | Hungary | Nédli et al., 2014 |
| 5 | *Moina* cf. *brachiata* clade E | Moina brachiata Mbra1HU4 | JN641832.1 | Hungary | Nédli et al., 2014 |
| 5 | *Moina* cf. *brachiata* clade E | Moina brachiata Mbra18HU | JN641838.1 | Hungary | Nédli et al., 2014 |
| 5 | *Moina* cf. *brachiata* clade E | Moina brachiata MbraHU2 | JN641839.1 | Hungary | Nédli et al., 2014 |
| 5 | *Moina* cf. *brachiata* clade E | Moina brachiata MbraHU4 | JN641841.1 | Hungary | Nédli et al., 2014 |
| 5 | *Moina* cf. *brachiata* clade E | Moina brachiata Mbra1HU5 | JN641843.1 | Hungary | Nédli et al., 2014 |
| 5 | *Moina* cf. *brachiata* clade E | Moina brachiata Mbra1HU6 | JN641845.1 | Hungary | Nédli et al., 2014 |
| 5 | *Moina* cf. *brachiata* clade E | Moina brachiata Mbra1HU7 | JN641850.1 | Hungary | Nédli et al., 2014 |
| 5 | *Moina* cf. *brachiata* clade E | Moina brachiata Mbra1HU8 | JN641851.1 | Hungary | Nédli et al., 2014 |
| 5 | *Moina* cf. *brachiata* clade E | Moina brachiata Mbra1HU9 | JN641852.1 | Hungary | Nédli et al., 2014 |
| 5 | *Moina* cf. *brachiata* clade E | Moina brachiata Mbra11HU | JN641854.1 | Hungary | Nédli et al., 2014 |
| 5 | *Moina* cf. *brachiata* clade E | Moina brachiata Mbra1HU11 | JN641857.1 | Hungary | Nédli et al., 2014 |
| 5 | *Moina* cf. *brachiata* clade E | Moina brachiata Mbra11HU12 | JN641858.1 | Hungary | Nédli et al., 2014 |
| 5 | *Moina* cf. *brachiata* clade E | Moina brachiata Mbra1HU13 | JN641859.1 | Hungary | Nédli et al., 2014 |
| 5 | *Moina* cf. *brachiata* clade E | Moina brachiata Mbra1HU14 | JN641860.1 | Hungary | Nédli et al., 2014 |
| 5 | *Moina* cf. *brachiata* clade E | Moina brachiata Mbra115HU | JN641812.1 | Hungary | Nédli et al., 2014 |
| 5 | *Moina* cf. *brachiata* clade E | Moina brachiata Mbra14HU1 | JN641813.1 | Hungary | Nédli et al., 2014 |
| 5 | *Moina* cf. *brachiata* clade E | Moina brachiata Mbra14HU2 | JN641814.1 | Hungary | Nédli et al., 2014 |
| 5 | *Moina* cf. *brachiata* clade E | Moina brachiata Mbra14HU3 | JN641815.1 | Hungary | Nédli et al., 2014 |
| 5 | *Moina* cf. *brachiata* clade E | Moina brachiata Mbra14HU4 | JN641825.1 | Hungary | Nédli et al., 2014 |
| 5 | *Moina* cf. *brachiata* clade E | Moina brachiata Mbra110HU1 | JN641821.1 | Hungary | Nédli et al., 2014 |
| 5 | *Moina* cf. *brachiata* clade E | Moina brachiata Mbra110HU2 | JN641822.1 | Hungary | Nédli et al., 2014 |
| 5 | *Moina* cf. *brachiata* clade E | Moina brachiata Mbra110HU | JN641844.1 | Hungary | Nédli et al., 2014 |
| 5 | *Moina* cf. *brachiata* clade E | Moina brachiata Mbra16HU | JN641827.1 | Hungary | Nédli et al., 2014 |
| 5 | *Moina* cf. *brachiata* clade E | Moina brachiata Mbra17HU | JN641828.1 | Hungary | Nédli et al., 2014 |
| 5 | *Moina* cf. *brachiata* clade E | Moina brachiata Mbra19HU | JN641831.1 | Hungary | Nédli et al., 2014 |
| 5 | *Moina* cf. *brachiata* clade E | Moina brachiata Mbra15HU | JN641853.1 | Hungary | Nédli et al., 2014 |
| 5 | *Moina* cf. *brachiata* clade E | Moina brachiata Mbra13HU | JN641855.1 | Hungary | Nédli et al., 2014 |
| 5 | *Moina* cf. *brachiata* clade E | Moina brachiata Mbra12HU | JN641863.1 | Hungary | Nédli et al., 2014 |
| 6 | *Moina* cf. *brachiata* clade F | Moina brachiata Mbra3HU | JN641861.1 | Hungary | Nédli et al., 2014 |
| 6 | *Moina* cf. *brachiata* clade F | Moina brachiata Mbra31HU | JN641862.1 | Hungary | Nédli et al., 2014 |
| 9 | *Moina* cf. *micrura* clade I | Moina micrura Kazan | HQ336797.1 | Russia (Tatartstan Autonomous Republic) | Frolova et al., direct submission |
| 9 | *Moina* cf. *micrura* clade I | Moina sp HE-643.1 SouthKorea | KC617390.1 | South Korea | Prosser et al., 2013 |
| 9 | *Moina* cf. *micrura* clade I | Moina sp HE-647.1 SouthKorea | KC617391.1 | South Korea | Prosser et al., 2013 |
| 9 | *Moina* cf. *micrura* clade I | Moina sp HE-645.1 SouthKorea | KC617392.1 | South Korea | Prosser et al., 2013 |
| 9 | *Moina* cf. *micrura* clade I | Moina sp HE-646.1 SouthKorea | KC617393.1 | South Korea | Prosser et al., 2013 |
| 9 | *Moina* cf. *micrura* clade I | Moina sp HE-644.1 SouthKorea | KC617394.1 | South Korea | Prosser et al., 2013 |
| 20 | *Moina* cf. *salina* clade N | Moina mongolica China | KP027211.1 | China | Chen et al., direct submission |
| 19 | *Moina macrocopa macrocopa* | Moina sp BOLDAAK6825 BIOUG<CANBrMoi000 | DQ889112.1 | Canada? | Costa et al., 1997 |
| 19 | *Moina macrocopa macrocopa* | Moina sp 1 JRdW-2005 | DQ310653.1 | Canada? | De Waard et al., 2006 |
| 19 | *Moina macrocopa macrocopa* | Moina macrocopa Hungary | JN657688.1 | Hungary | Nédli et al., 2014 |
| 19 | *Moina macrocopa macrocopa* | Moina macrocopa Hungary | JN657690.1 | Hungary | Nédli et al., 2014 |
| 19 | *Moina macrocopa macrocopa* | Moina macrocopa Hungary | JN657691.1 | Hungary | Nédli et al., 2014 |
| 19 | *Moina macrocopa macrocopa* | Moina macrocopa Hungary | JN657689.1 | Hungary | Nédli et al., 2014 |
| 13 | *Moina* cf*. micrura* 1 | Moina cf micrura 1 MEG-2008 ZPLMX363 | EU702200.1 | Mexico | Elías-Gutiérrez et al., 2008 |
| 13 | *Moina* cf. *micrura* 1 | Moina cf micrura 1 MEG-2008 ZPLMX290 | EU702203.1 | Mexico | Elías-Gutiérrez et al., 2008 |
| 13 | *Moina* cf. *micrura* 1 | Moina cf micrura 1 MEG-2008 ZPLMX288 | EU702204.1 | Mexico | Elías-Gutiérrez et al., 2008 |
| 13 | *Moina* cf. *micrura* 1 | Moina cf micrura 1 MEG-2008 ZPLMX287 | EU702205.1 | Mexico | Elías-Gutiérrez et al., 2008 |
| 13 | *Moina* cf. *micrura* 1 | Moina cf micrura 1 MEG-2008 ZPLMX286 | EU702206.1 | Mexico | Elías-Gutiérrez et al., 2008 |
| 13 | *Moina* cf. *micrura* 1 | Moina cf micrura 1 MEG-2008 ZPLMX285 | EU702207.1 | Mexico | Elías-Gutiérrez et al., 2008 |
| 13 | *Moina* cf. *micrura* 1 | Moina cf micrura 1 MEG-2008 ZPLMX376 | EU702208.1 | Mexico | Elías-Gutiérrez et al., 2008 |
| 13 | *Moina* cf. *micrura* 1 | Moina cf micrura 1 MEG-2008 ZPLMX375 | EU702209.1 | Mexico | Elías-Gutiérrez et al., 2008 |
| 13 | *Moina* cf. *micrura* 1 | Moina cf micrura 1 MEG-2008 ZPLMX374 | EU702210.1 | Mexico | Elías-Gutiérrez et al., 2008 |
| 13 | *Moina* cf. *micrura* 1 | Moina cf micrura 1 MEG-2008 ZPLMX373 | EU702211.1 | Mexico | Elías-Gutiérrez et al., 2008 |
| 13 | *Moina* cf. *micrura* 1 | Moina cf micrura 1 MEG-2008 ZPLMX372 | EU702212.1 | Mexico | Elías-Gutiérrez et al., 2008 |
| 13 | *Moina* cf. *micrura* 1 | Moina cf micrura 1 MEG-2008 ZPLMX371 | EU702213.1 | Mexico | Elías-Gutiérrez et al., 2008 |
| 13 | *Moina* cf. *micrura* 1 | Moina cf micrura 1 MEG-2008 ZPLMX369 | EU702215.1 | Mexico | Elías-Gutiérrez et al., 2008 |
| 13 | *Moina* cf. *micrura* 1 | Moina cf micrura 1 MEG-2008 ZPLMX368 | EU702216.1 | Mexico | Elías-Gutiérrez et al., 2008 |
| 13 | *Moina* cf. *micrura* 1 | Moina cf micrura 1 MEG-2008 ZPLMX367 | EU702217.1 | Mexico | Elías-Gutiérrez et al., 2008 |
| 13 | *Moina* cf. *micrura* 1 | Moina cf micrura 1 MEG-2008 ZPLMX366 | EU702218.1 | Mexico | Elías-Gutiérrez et al., 2008 |
| 13 | *Moina* cf. *micrura* 1 | Moina cf micrura 1 MEG-2008 ZPLMX168 | EU702226.1 | Mexico | Elías-Gutiérrez et al., 2008 |
| 13 | *Moina* cf. *micrura* 1 | Moina cf micrura 1 MEG-2008 ZPLMX167 | EU702227.1 | Mexico | Elías-Gutiérrez et al., 2008 |
| 13 | *Moina* cf. *micrura* 1 | Moina cf micrura 1 MEG-2008 ZPLMX370 | EU702214.1 | Mexico | Elías-Gutiérrez et al., 2008 |
| 15 | *Moina* cf. *micrura* 2 | Moina cf micrura 2 MEG-2008 ZPLMX348 | EU702228.1 | Mexico | Elías-Gutiérrez et al., 2008 |
| 15 | *Moina* cf. *micrura* 2 | Moina cf micrura 2 MEG-2008 ZPLMX347 | EU702229.1 | Mexico | Elías-Gutiérrez et al., 2008 |
| 15 | *Moina* cf. *micrura* 2 | Moina cf micrura 2 MEG-2008 ZPLMX346 | EU702230.1 | Mexico | Elías-Gutiérrez et al., 2008 |
| 15 | *Moina* cf. *micrura* 2 | Moina cf micrura 2 MEG-2008 ZPLMX345 | EU702231.1 | Mexico | Elías-Gutiérrez et al., 2008 |
| 15 | *Moina* cf. *micrura* 2 | Moina cf micrura 2 MEG-2008 ZPLMX344 | EU702232.1 | Mexico | Elías-Gutiérrez et al., 2008 |
| 15 | *Moina* cf. *micrura* 2 | Moina cf micrura 2 MEG-2008 ZPLMX343 | EU702233.1 | Mexico | Elías-Gutiérrez et al., 2008 |
| 15 | *Moina* cf. *micrura* 2 | Moina cf micrura 2 MEG-2008 ZPLMX342 | EU702234.1 | Mexico | Elías-Gutiérrez et al., 2008 |
| 15 | *Moina* cf. *micrura* 2 | Moina cf micrura 2 MEG-2008 ZPLMX341 | EU702235.1 | Mexico | Elías-Gutiérrez et al., 2008 |
| 15 | *Moina* cf. *micrura* 2 | Moina cf micrura 2 MEG-2008 ZPLMX340 | EU702236.1 | Mexico | Elías-Gutiérrez et al., 2008 |
| 15 | *Moina* cf. *micrura* 2 | Moina cf micrura 2 MEG-2008 ZPLMX339 | EU702237.1 | Mexico | Elías-Gutiérrez et al., 2008 |
| 15 | *Moina* cf. *micrura* 2 | Moina cf micrura 2 MEG-2008 ZPLMX338 | EU702238.1 | Mexico | Elías-Gutiérrez et al., 2008 |
| 15 | *Moina* cf. *micrura* 2 | Moina cf micrura 2 MEG-2008 ZPLMX188 | EU702239.1 | Mexico | Elías-Gutiérrez et al., 2008 |
| 15 | *Moina* cf. *micrura* 2 | Moina cf micrura 2 MEG-2008 ZPLMX153 | EU702240.1 | Mexico | Elías-Gutiérrez et al., 2008 |
| 15 | *Moina* cf. *micrura* 2 | Moina cf micrura 2 MEG-2008 ZPLMX154 | EU702241.1 | Mexico | Elías-Gutiérrez et al., 2008 |
| 15 | *Moina* cf. *micrura* 2 | Moina cf micrura 2 MEG-2008 ZPLMX147 | EU702242.1 | Mexico | Elías-Gutiérrez et al., 2008 |
| 15 | *Moina* cf. *micrura* 2 | Moina cf micrura 2 MEG-2008-619.1 | KC617395.1 | Mexico | Elías-Gutiérrez et al., 2008 |
| 15 | *Moina* cf. *micrura* 2 | Moina cf micrura 2 MEG-2008-619 | KC617396.1 | Mexico | Elías-Gutiérrez et al., 2008 |
| 15 | *Moina* cf. *micrura* 2 | Moina cf micrura 2 MEG-2008 AS30a1 | KC617104.1 | Mexico | Elías-Gutiérrez et al., 2008 |
| 15 | *Moina* cf. *micrura* 2 | Moina cf micrura 2 MEG-2008-148 | KC617106.1 | Mexico | Elías-Gutiérrez et al., 2008 |
| 15 | *Moina* cf. *micrura* 2 | Moina cf micrura 2 MEG-2008 AS49a1 | KC617108.1 | Mexico | Elías-Gutiérrez et al., 2008 |
| 15 | *Moina* cf. *micrura* 2 | Moina cf micrura 2 MEG-2008-147A | KC617114.1 | Mexico | Elías-Gutiérrez et al., 2008 |
| 15 | *Moina* cf. *micrura* 2 | Moina cf micrura 2 MEG-2008-148A | KC617115.1 | Mexico | Elías-Gutiérrez et al., 2008 |
| 15 | *Moina* cf. *micrura* 2 | Moina cf micrura 2 MEG-2008-149A | KC617116.1 | Mexico | Elías-Gutiérrez et al., 2008 |
| 15 | *Moina* cf. *micrura* 2 | Moina cf micrura 2 MEG-2008-405 | KC617697.1 | Mexico | Elías-Gutiérrez et al., 2008 |
| 15 | *Moina* cf. *micrura* 2 | Moina cf micrura 2 MEG-2008-425.1 | KC617698.1 | Mexico | Elías-Gutiérrez et al., 2008 |
| 15 | *Moina* cf. *micrura* 2 | Moina cf micrura 2 MEG-2008-218.1 | KC617699.1 | Mexico | Elías-Gutiérrez et al., 2008 |
| 15 | *Moina* cf. *micrura* 2 | Moina cf micrura 2 MEG-2008-220.1 | KC617700.1 | Mexico | Elías-Gutiérrez et al., 2008 |
| 15 | *Moina* cf. *micrura* 2 | Moina cf micrura 2 MEG-2008-407.1 | KC617701.1 | Mexico | Elías-Gutiérrez et al., 2008 |
| 15 | *Moina* cf. *micrura* 2 | Moina cf micrura 2 MEG-2008-406.1 | KC617702.1 | Mexico | Elías-Gutiérrez et al., 2008 |
| 15 | *Moina* cf. *micrura* 2 | Moina cf micrura 2 MEG-2008-405.1 | KC617703.1 | Mexico | Elías-Gutiérrez et al., 2008 |
| 15 | *Moina* cf. *micrura* 2 | Moina cf micrura 2 MEG-2008-278.1 | KC617705.1 | Mexico | Elías-Gutiérrez et al., 2008 |
| 15 | *Moina* cf. *micrura* 2 | Moina cf micrura 2 MEG-2008-279.1 | KC617706.1 | Mexico | Elías-Gutiérrez et al., 2008 |
| 15 | *Moina* cf. *micrura* 2 | Moina cf micrura 2 MEG-2008-377.1 | KC617709.1 | Mexico | Elías-Gutiérrez et al., 2008 |
| 11 | *Moina* cf. *micrura* 5 | Moina cf micrura 5 MEG-2013-566.1 | KC617397.1 | Mexico | Prosser et al., 2013 |
| 11 | *Moina* cf. *micrura* 5 | Moina cf micrura 5 MEG-2013-565.1 | KC617398.1 | Mexico | Prosser et al., 2013 |
| 11 | *Moina* cf. *micrura* 5 | Moina cf micrura 5 MEG-2013-564.1 | KC617399.1 | Mexico | Prosser et al., 2013 |
| 11 | *Moina* cf. *micrura* 5 | Moina cf micrura 5 MEG-2013-563.1 | KC617400.1 | Mexico | Prosser et al., 2013 |
| 11 | *Moina* cf. *micrura* 5 | Moina cf micrura 5 MEG-2013-601.1 | KC617401.1 | Mexico | Prosser et al., 2013 |
| 11 | *Moina* cf. *micrura* 5 | Moina cf micrura 5 MEG-2013-601 | KC617402.1 | Mexico | Prosser et al., 2013 |
| 11 | *Moina* cf. *micrura* 5 | Moina cf micrura 5 MEG-2013-563 | KC617403.1 | Mexico | Prosser et al., 2013 |
| 11 | *Moina* cf. *micrura* 5 | Moina cf micrura 5 MEG-2013-564 | KC617404.1 | Mexico | Prosser et al., 2013 |
| 11 | *Moina* cf. *micrura* 5 | Moina cf micrura 5 MEG-2013-565 | KC617405.1 | Mexico | Prosser et al., 2013 |
| 11 | *Moina* cf. *micrura* 5 | Moina cf micrura 5 MEG-2013-566 | KC617406.1 | Mexico | Prosser et al., 2013 |
| 11 | *Moina* cf. *micrura* 5 | Moina cf micrura 5 MEG-2013-567.1 | KC617407.1 | Mexico | Prosser et al., 2013 |
| 11 | *Moina* cf. *micrura* 5 | Moina cf micrura 5 MEG-2013-252 | KC617124.1 | Mexico | Prosser et al., 2013 |
| 11 | *Moina* cf. *micrura* 5 | Moina cf micrura 5 MEG-2013-253 | KC617125.1 | Mexico | Prosser et al., 2013 |
| 11 | *Moina* cf. *micrura* 5 | Moina cf micrura 5 MEG-2013-251.1 | KC617714.1 | Mexico | Prosser et al., 2013 |
| 11 | *Moina* cf. *micrura* 5 | Moina cf micrura 5 MEG-2013-252.1 | KC617715.1 | Mexico | Prosser et al., 2013 |
| 11 | *Moina* cf. *micrura* 5 | Moina cf micrura 5 MEG-2013-253.1 | KC617716.1 | Mexico | Prosser et al., 2013 |
| 11 | *Moina* cf. *micrura* 5 | Moina cf micrura 5 MEG-2013-304.1 | KC617717.1 | Mexico | Prosser et al., 2013 |
| 15 | *Moina* cf. *micrura* 2 | Moina cf micrura 2 MEG-2008 AS53a1 | KC617105.1 | Mexico | Elías-Gutiérrez et al., 2008 |
| 15 | *Moina* cf. *micrura* 2 | Moina cf micrura 2 MEG-2008 AS30a2 | KC617112.1 | Mexico | Elías-Gutiérrez et al., 2008 |
| 17 | *Moina* *macrocopa* *amiricana* | Moina macrocopa ZMXII-568 | KC617129.1 | Mexico | Prosser et al., 2013 |
| 17 | *Moina* *macrocopa* *amiricana* | Moina macrocopa ZMXII-649 | KC617131.1 | Mexico | Prosser et al., 2013 |
| 17 | *Moina* *macrocopa* *amiricana* | Moina macrocopa ZMXII-637 | KC617132.1 | Mexico | Prosser et al., 2013 |
| 17 | *Moina* *macrocopa* *amiricana* | Moina macrocopa ZMXII-625 | KC617133.1 | Mexico | Prosser et al., 2013 |
| 17 | *Moina* *macrocopa* *amiricana* | Moina macrocopa ZMXII-613 | KC617134.1 | Mexico | Prosser et al., 2013 |
| 17 | *Moina* *macrocopa* *amiricana* | Moina macrocopa AS22a3 | KC617130.1 | Mexico | Prosser et al., 2013 |
| 12 | *Moina* cf. *micrura* 4 | Moina cf micrura 4 MEG-2013-249.1 | KC617710.1 | Mexico | Prosser et al., 2013 |
| 12 | *Moina* cf. *micrura* 4 | Moina cf micrura 4 MEG-2013-248.1 | KC617711.1 | Mexico | Prosser et al., 2013 |
| 12 | *Moina* cf. *micrura* 4 | Moina cf micrura 4 MEG-2013-246.1 | KC617712.1 | Mexico | Prosser et al., 2013 |
| 12 | *Moina* cf. *micrura* 4 | Moina cf micrura 4 MEG-2013-250.1 | KC617713.1 | Mexico | Prosser et al., 2013 |
| 14 | *Moina* sp. | Moina sp 2 JRdW-2005 | DQ310654.1 | Canada? | De Waard et al., 2006 |
| 17 | *Moina* *macrocopa* *amiricana* | Moina macrocopa ZPLMX335 | EU702246.1 | Mexico | Elías-Gutiérrez et al., 2008 |
| 17 | *Moina* *macrocopa* *amiricana* | Moina macrocopa ZPLMX334 | EU702247.1 | Mexico | Elías-Gutiérrez et al., 2008 |
| 17 | *Moina* *macrocopa* *amiricana* | Moina macrocopa ZPLMX262 | EU702249.1 | Mexico | Elías-Gutiérrez et al., 2008 |
