# Supplementary material for: DNA Barcoding Reveals High Cryptic Diversity in the North Eurasian Moina Species (Crustacea: Cladocera)
Source: PLoS One. 2016 Aug 24;11(8):e0161737. doi: 10.1371/journal.pone.0161737 (PMC4996527; doi:10.1371/journal.pone.0161737)
Supplement: S3 Table — Number of phylogroups correspond to thise in other tables. Standard error estimates are shown above the diagonal and were obtained by a bootstrap procedure (10000 replicates). Analyses were conducted using the Tamura 3-parameter model. The rate variation among sites was modeled with a gamma distribution. All ambiguous positions were removed for each sequence pair. (DOC) [file pone.0161737.s003.doc]

**S3 Table.** Intra-group and inter-group genetic distances of the 21 groups involved 657 nucleotide sequences of *Moina*. Number of phylogroups correspond to thise in other tables. Standard error estimates are shown above the diagonal and were obtained by a bootstrap procedure (10000 replicates). Analyses were conducted using the Tamura 3-parameter model. The rate variation among sites was modeled with a gamma distribution. All ambiguous positions were removed for each sequence pair.

|  | **01** | **02** | **03** | **04** | **05** | **06** | **07** | **08** | **09** | **10** | **11** | **12** | **13** | **14** | **15** | **16** | **17** | **18** | **19** | **20** | **21** |
| --- | --- | --- | --- | --- | --- | --- | --- | --- | --- | --- | --- | --- | --- | --- | --- | --- | --- | --- | --- | --- | --- |
| **01** |  | 0.009 | 0.016 | 0.017 | 0.019 | 0.018 | 0.021 | 0.024 | 0.022 | 0.024 | 0.023 | 0.023 | 0.023 | 0.021 | 0.023 | 0.026 | 0.023 | 0.023 | 0.026 | 0.030 | 0.029 |
| **02** | 0.048 |  | 0.017 | 0.019 | 0.020 | 0.019 | 0.023 | 0.023 | 0.023 | 0.025 | 0.024 | 0.024 | 0.025 | 0.023 | 0.023 | 0.027 | 0.024 | 0.022 | 0.027 | 0.027 | 0.028 |
| **03** | 0.116 | 0.117 |  | 0.019 | 0.022 | 0.017 | 0.019 | 0.024 | 0.023 | 0.026 | 0.024 | 0.025 | 0.024 | 0.021 | 0.021 | 0.032 | 0.023 | 0.022 | 0.026 | 0.027 | 0.028 |
| **04** | 0.116 | 0.131 | 0.141 |  | 0.008 | 0.018 | 0.022 | 0.025 | 0.020 | 0.022 | 0.019 | 0.020 | 0.022 | 0.022 | 0.024 | 0.027 | 0.021 | 0.022 | 0.025 | 0.028 | 0.027 |
| **05** | 0.128 | 0.136 | 0.156 | 0.039 |  | 0.020 | 0.023 | 0.029 | 0.022 | 0.023 | 0.022 | 0.022 | 0.024 | 0.023 | 0.027 | 0.027 | 0.025 | 0.024 | 0.027 | 0.030 | 0.030 |
| **06** | 0.137 | 0.142 | 0.126 | 0.128 | 0.142 |  | 0.019 | 0.023 | 0.019 | 0.020 | 0.021 | 0.022 | 0.021 | 0.019 | 0.020 | 0.029 | 0.021 | 0.020 | 0.021 | 0.028 | 0.029 |
| **07** | 0.158 | 0.181 | 0.150 | 0.180 | 0.184 | 0.152 |  | 0.021 | 0.020 | 0.023 | 0.024 | 0.026 | 0.021 | 0.020 | 0.023 | 0.027 | 0.020 | 0.021 | 0.027 | 0.029 | 0.029 |
| **08** | 0.189 | 0.191 | 0.202 | 0.208 | 0.232 | 0.192 | 0.160 |  | 0.022 | 0.024 | 0.023 | 0.023 | 0.022 | 0.021 | 0.022 | 0.027 | 0.023 | 0.022 | 0.028 | 0.032 | 0.034 |
| **09** | 0.177 | 0.182 | 0.195 | 0.163 | 0.170 | 0.159 | 0.168 | 0.177 |  | 0.022 | 0.020 | 0.021 | 0.018 | 0.020 | 0.021 | 0.026 | 0.020 | 0.020 | 0.024 | 0.028 | 0.027 |
| **10** | 0.198 | 0.208 | 0.205 | 0.179 | 0.181 | 0.161 | 0.189 | 0.195 | 0.185 |  | 0.019 | 0.020 | 0.022 | 0.021 | 0.021 | 0.024 | 0.025 | 0.026 | 0.027 | 0.030 | 0.032 |
| **11** | 0.185 | 0.195 | 0.206 | 0.155 | 0.172 | 0.170 | 0.190 | 0.189 | 0.157 | 0.150 |  | 0.009 | 0.017 | 0.016 | 0.018 | 0.023 | 0.022 | 0.024 | 0.022 | 0.031 | 0.032 |
| **12** | 0.188 | 0.193 | 0.208 | 0.158 | 0.174 | 0.181 | 0.215 | 0.202 | 0.171 | 0.161 | 0.048 |  | 0.017 | 0.018 | 0.019 | 0.025 | 0.021 | 0.023 | 0.022 | 0.027 | 0.028 |
| **13** | 0.193 | 0.211 | 0.202 | 0.173 | 0.194 | 0.173 | 0.168 | 0.183 | 0.145 | 0.175 | 0.125 | 0.133 |  | 0.017 | 0.017 | 0.027 | 0.021 | 0.021 | 0.022 | 0.028 | 0.028 |
| **14** | 0.175 | 0.195 | 0.187 | 0.182 | 0.184 | 0.155 | 0.166 | 0.175 | 0.169 | 0.174 | 0.116 | 0.138 | 0.123 |  | 0.011 | 0.024 | 0.021 | 0.018 | 0.023 | 0.029 | 0.030 |
| **15** | 0.185 | 0.192 | 0.169 | 0.195 | 0.220 | 0.159 | 0.182 | 0.173 | 0.164 | 0.174 | 0.133 | 0.141 | 0.129 | 0.071 |  | 0.026 | 0.018 | 0.018 | 0.022 | 0.029 | 0.030 |
| **16** | 0.218 | 0.231 | 0.271 | 0.224 | 0.226 | 0.237 | 0.223 | 0.232 | 0.212 | 0.211 | 0.198 | 0.214 | 0.233 | 0.201 | 0.213 |  | 0.024 | 0.024 | 0.024 | 0.032 | 0.031 |
| **17** | 0.191 | 0.199 | 0.189 | 0.182 | 0.204 | 0.176 | 0.165 | 0.194 | 0.155 | 0.214 | 0.185 | 0.181 | 0.168 | 0.170 | 0.145 | 0.201 |  | 0.012 | 0.018 | 0.026 | 0.026 |
| **18** | 0.182 | 0.184 | 0.185 | 0.184 | 0.196 | 0.172 | 0.163 | 0.189 | 0.159 | 0.225 | 0.188 | 0.190 | 0.171 | 0.148 | 0.136 | 0.193 | 0.069 |  | 0.018 | 0.029 | 0.029 |
| **19** | 0.211 | 0.230 | 0.222 | 0.205 | 0.223 | 0.173 | 0.225 | 0.230 | 0.204 | 0.225 | 0.181 | 0.190 | 0.183 | 0.190 | 0.182 | 0.192 | 0.145 | 0.137 |  | 0.026 | 0.028 |
| **20** | 0.239 | 0.223 | 0.220 | 0.233 | 0.240 | 0.232 | 0.233 | 0.248 | 0.228 | 0.239 | 0.254 | 0.238 | 0.226 | 0.245 | 0.238 | 0.262 | 0.212 | 0.241 | 0.222 |  | 0.013 |
| **21** | 0.238 | 0.235 | 0.240 | 0.221 | 0.245 | 0.245 | 0.243 | 0.281 | 0.227 | 0.258 | 0.268 | 0.249 | 0.235 | 0.251 | 0.257 | 0.264 | 0.218 | 0.243 | 0.253 | 0.085 |  |
